# Massively parallel microbubble nano-assembly

**DOI:** 10.1038/s41467-025-62070-9

**Published:** 2025-07-22

**Authors:** Hyungmok Joh, Bin Lian, Shaw-iong Hsueh, Zhichao Ma, Keng-Jung Lee, Si-Yang Zheng, Peer Fischer, Donglei Emma Fan

**Affiliations:** 1 Materials Science and Engineering Program, Texas Materials Institute, The University of Texas at Austin, Austin, TX USA; 2 Chandra Family Department of Electrical and Computer Engineering, The University of Texas at Austin, Austin, TX USA; 3 Max Planck Institute for Intelligent Systems, Stuttgart, Germany; 4 Biomedical Engineering, Carnegie Mellon University, Pittsburgh, PA USA; 5 Electrical & Computer Engineering, Carnegie Mellon University, Pittsburgh, PA USA; 6 Max Planck Institute for Medical Research, Heidelberg, Germany; 7 Institute for Molecular Systems Engineering and Advanced Materials, Heidelberg University, Heidelberg, Germany; 8 Center for Nanomedicine, Institute for Basic Science (IBS), Seoul, Republic of Korea; 9 Department of Nano Biomedical Engineering (NanoBME),Advanced Science Institute, Yonsei University, Seoul, Republic of Korea; 10 Walker Department of Mechanical Engineering, University of Texas at Austin, Austin, TX USA; 11 Present Address: School of Biomedical Engineering and Institute of Medical Robotics, Shanghai Jiao Tong University, Shanghai, China

**Keywords:** Surface patterning, Nanofabrication and nanopatterning, Biosensors

## Abstract

Microbubbles are an important tool due to their unique mechanical, acoustic, and dynamical properties. Yet, it remains challenging to generate microbubbles quickly in a parallel, biocompatible, and controlled manner. Here, we present an opto-electrochemical method that combines precise light-based projection with low-energy electrolysis, realizing defined microbubble patterns that in turn trigger assembly processes. The size of the bubbles can be controlled from a few to over hundred micrometers with a spatial accuracy of ~2 μm. The minimum required light intensity is only ~0.1 W/cm^2^, several orders of magnitude lower compared to other light-enabled methods. We demonstrate the assembly of prescribed patterns of 40-nm nanocrystals, 200 nm extracellular vesicles, polymer nanospheres, and live bacteria. We show how nanosensor-bacterial-cell arrays can be formed for spectroscopic profiling of metabolites and antibiotic response of bacterial assemblies. The combination of a photoconductor with electrochemical techniques enables low-energy, low-temperature bubble generation, advantageous for large-scale, one-shot patterning of diverse particles in a biocompatible manner. The microbubble-platform is highly versatile and promises new opportunities in nanorobotics, nanomanufacturing, high-throughput bioassays, single cell omics, bioseparation, and drug screening and discovery.

## Abstract (web summary)

Rapid, biocompatible, and parallel generation of microbubbles remains a major challenge despite their high potential in biomedical and materials applications. The authors introduce a low-energy, biocompatible opto-electrochemical platform that enables precise, parallel microbubble patterning for programmable assembly of nanoscale to biological materials with high spatial accuracy.

## Introduction

Microbubbles exhibit special mechanical, acoustic, and dynamical properties and have become important tools in fields ranging from medical imaging to microfabrication. Their ability to interact with soft matter has been explored for diverse applications, including microfluidic pumping and mixing^1–5^, chemical switching^6,7^, drug delivery^8,9^, shaping ultrasound fields^10^, and in the propulsion of micro/nanorobots^11–14^. Ultrasonically excited microbubbles can transduce forces directly^15^, or indirectly via streaming or nonlinear effects, and can be amplified to move objects in solution^16^. Microbubbles that are not resonantly excited can induce local fluid flows to manipulate colloidal and nano-particulate matter^17^. This capability is particularly useful in overcoming the technical challenges in complex device design and fabrication for high-throughput bioassays, single-cell omics, and rapid drug screening. Technically, microbubble-enabled particle assembly often involves the generation of a single microbubble with a focused laser beam to deposit particles onto a substrate via convective flows that form around the microbubble. The laser beam can also be raster-scanned to move the micro-bubble and thereby drive complex nanoparticle deposits^17^. However, this method is relatively slow as it involves a single microbubble and a major drawback is that it operates at elevated temperatures as the microbubbles are thermo-optically generated, which severely restricts its usefulness for any potential biological application.

Conventionally, microbubbles are formed in prefabricated templates, including concave microstructures, which can then be used to mediate acoustic forces^14^. However, the bubble patterns are defined by the template and are thus static. Alternately, the use of laser-induced heating can form opto-thermal microbubbles at the focus of the laser. While the use of plasmonic Au/Ag nanoparticles or surfaces reduces the required laser power^18–20^, the direct formation of a microbubble with a laser always requires relatively high intensities and temperatures (>60° C up to 250° C, depending on bubble size^17^). As the opto-thermal approach utilizes a laser focus, it is difficult to adapt this method for larger-scale applications and importantly restricts the capability in working with temperature-sensitive biological samples. Therefore, it is desirable to explore alternative schemes to form bubbles, such as electrolysis. Thus far, there have been no reports on biologically meaningful applications (with the exception of cell lysis) using thermal bubbles^21^. While electrolysis can proceed at room temperature, it is challenging to define the size, location, or number of bubbles that are generated. Overall, several drawbacks of existing state-of-the-art techniques restrict practical applications of microbubbles (Table S1)^22,23^.

Here, we show an alternative microbubble scheme that uses low-intensity light to obtain defined microbubble patterns and we show that these can be used to assemble diverse colloidal matter, including extracellular vesicles and biological cells at room temperature, into complex patterns. The term “assembly” here describes primarily the action of collecting and aggregating entities without ordering them, but in some cases, we did observe the formation of more ordered semicrystalline arrangements of particles after bubble deposition.

We show that very low light levels can trigger the formation of microbubbles via the hydrogen evolution reaction (HER). We utilize a photoconducting α-Si:H surface deposited on a fluorine-doped tin oxide (FTO) substrate to convert low-intensity light patterns into versatile bubble patterns. In the absence of light, the α-Si:H prevents any current from flowing and hence prevents the gas formation via electrolysis. However, electrolysis proceeds when the α-Si:H becomes conducting under light, even at low intensities (~0.1 W/cm^2^). This enables room temperature applications and ensures the high scalability of our technique, as a digital light projector (DLP) can project entire images and thus form complex bubble patterns “in one shot” made of many bubbles simultaneously, opposed to serial, single bubble-based techniques. We would like to stress that the light intensity is several orders of magnitude lower compared to that required by various other light-based bubble techniques^19,24^. Furthermore, standard electrolysis results in the formation of clusters of microbubbles in an activated region, but we show that the addition of nanoparticles can prevent the formation of small satellite bubbles, such that one can, for the first time, form individually light-addressable bubbles from a few to hundreds of µm in diameter.

Next, we demonstrate that the bubbles also drive the assembly of particles in the suspension, which, in contrast to opto-thermally generated bubbles, does not rely on thermocapillary or thermal convection currents. Rather, the attraction of particles to the bubble surface can be attributed to a surface tension and concentration gradient near a shrinking bubble^25^. The microbubble patterns are shown to be versatile for assembly from metallic and polymer nanoparticles to extracellular vesicles, cells, and particle-cell hybrids. Finally, as a proof-of-concept, we demonstrate a microbubble-enabled nanosensor assay. The technology not only permits integration with bioassays but also enables the dynamic monitoring of bacterial cell activity in response to drugs in quasi-real time.

## Results

### Working mechanism, characterization, and optimization

**Fig. 1:**
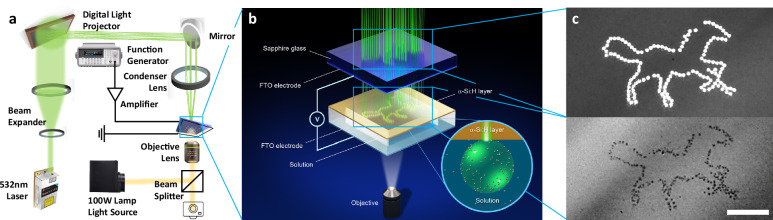
Experimental setup for bubble patterning. **a** Schematic of the DLP device used for large-area bubble patterning. **b** Close-up of the setup during bubble generation and subsequent particle patterning. **c** Laser pattern of a horse (top) and corresponding bubble pattern (bottom). Scale bar: 100 μm.

The experimental setup consists of a DLP device, which, illuminated by a 532 nm continuous wave laser, projects images onto a photoconductive *α*-Si:H substrate, where a PDMS well houses a sodium sulfate (Na_2_SO_4_)/nanoparticle solution and covered with a second FTO substrate as the counter electrode (Fig. 1a, b). An electric signal is applied, which drives the rapid formation of individual or large arrays of microbubbles at the projected light points (Fig. 1c). The application of both light and electric voltages is essential for the process; therefore, it is important to elucidate the working mechanism that generates microbubbles. We first determine the minimum required light intensity (MRLI) for the formation of bubbles.

**Fig. 2:**
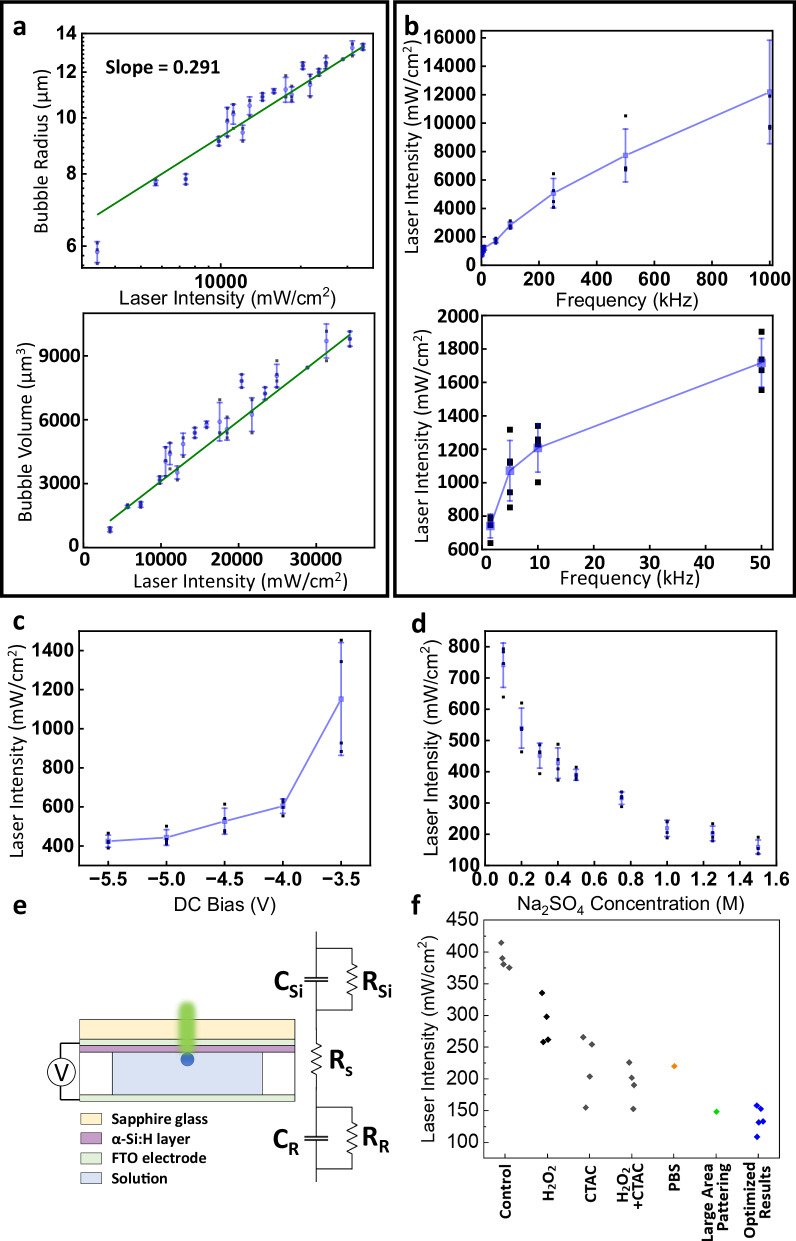
Characterization of bubble generation. **a** (top) Bubble radius linearly increases with laser intensity. (bottom) bubble volume vs laser intensity. Minimum required laser intensity as a function of **b** AC frequency, **c** DC bias, and **d** Na_2_SO_4_ electrolyte concentration. Unless stated otherwise, conditions used in characterization: 0.1 M Na2SO4 solution, −5 V DC/12 *V*pp at 1.5 kHz AC E-field applied for ~225 ms, and 500 μm PDMS well. **e** Schematic of the overall setup and corresponding equivalent circuit. **f** Optimization of the limit of light intensity for bubble generation. Conditions: (Black) 0.5 M Na_2_SO_4_, −5 V, 12 *V*_pp_ at 1.5 kHz, 100 ms, PDMS 500 µm. (Orange) PBS solution, −9 V, 2*V*_pp_ at 250 Hz, 200 ms, PDMS 100 µm. (Green) 0.75 M Na_2_SO_4_, −5 V, 10 V_pp_ at 1.5 kHz, PMDS 100 µm. (Blue) 1.5 M Na_2_SO_4_ with CTAC and H_2_O_2_, −12 V DC pulse with 50% duty cycle, 150 ms, PDMS 257 µm. Error bars: standard deviation (s.d.). Source data are provided as a Source Data file.

Upon light illumination with an applied electric field, a microbubble quickly forms. The radius (*r*) increases from ~6 to ~13 μm as the light intensity increases in an E-field (*V*_bias_: −5 V, *V*_pp_: 12 V at 1.5 kHz, 0.1 M Na_2_SO_4_ concentration) (Fig. 2a**)**; the power coefficient of 0.29 indicates the close-to-linear dependence of the bubble volume (*V*) on light intensity (Inset, Fig. 2a). The MRLI depends on the AC frequency and decreases from 12 to 0.74 W/cm^2^ when the AC frequency is lowered from 1 to 1.5 kHz (*V*_bias_: −5 V, *V*_pp_: 12 V, 0.1 M Na_2_SO_4_ concentration) (Fig. 2b). At a given AC *E*-field (*V*_pp_: 12 V at 1.5 kHz, 0.1 M Na_2_SO_4_ concentration), the MRLI is also reduced ~2.72-fold with an increasing superimposed DC bias from −3.5 to −5.5 V (Fig. 2c). Optionally, 0.1 M Na_2_SO_4_ has been used here to facilitate the bubble formation, but as shown below with phospahte-buffered saline (PBS) solutions, this is not essential. It should be also noted that a constant positive DC bias up to 9 V (or a positive bias superimposed on a symmetric AC voltage) does not generate bubbles, which suggest that the α-Si:H film conducts electrons under a negative bias, similar to p-type Si, supported by a voltage sweep test from +4 to −4 V (Fig. S1). Increasing the electric conductivity of the electrolyte solution also promotes bubble generation, where the MRLI is lowered by over 4.5-fold from 7.4 to 0.16 W/cm^2^ (*V*_bias_: −5 V, *V*_pp_: 12 V at 1.5 kHz) with the increase of Na_2_SO_4_ concentration from 0.1 to 1.5 M (Fig. 2d).

The requirement of light and electric voltage suggests three possible bubble-creation mechanisms: (1) ohmic heating of the light-illuminated *α*-Si:H, (2) light-based thermal heating, and (3) light-controlled electrochemical hydrolysis. While thermal heating has been widely explored for making microbubbles^20^, this working mechanism requires a light intensity that is at least 5 orders of magnitude higher than in our system^26^. Further calculation and numerical simulation indicate that neither ohmic heating nor thermal heating can account for the observed bubble creation, the effects of which only increase the temperature by «1 K (SI Note 1 and Fig. S2).

The electrochemical-reaction mechanism, in contrast, explains the observed bubble evolution and dependence on the laser intensity, the AC frequency, the DC bias, and the electrolyte concentration very well (Fig. 2a–d). For instance, the volume increases linearly with the light intensity (inset, Fig. 2a), which is in agreement with the linear dependence of the photocurrent (*i*_*p*_) (of the *α*-Si:H substrate) participating in electrochemical reaction, with photon rate (*N*_*0*_) and thus light, as shown in the following Eq. (1)^27^:

1 $${i}_{p}=e{N}_{0}\left(1-R\right)\left[1-\exp \left(-\alpha d\right)\right]{{\rm{\eta }}}{{\rm{\tau }}}/{t}_{t}\,,$$

where *R*, *α*, *d*, *η*, *τ*, *and t*_*t*_ are the surface reflection, surface absorption coefficient and depth, electron-hole pair generation efficiency, recombination lifetime, and carrier transit time, respectively.

We further model the system as an equivalent electrochemical circuit, which consists of an electric double layer (EDL) with a capacitance of *C*_*Si*_, next to the photoconductive Si electrode with a resistance of *R*_*Si*_, an electrolyte solution with a resistance of *R*_*s*_, and a second EDL next to the FTO counter electrode (*C*_*R*_) (Fig. 2e). Here, light turns on/off this electrochemical circuit path via controlling the Si-film’s local resistance. When light is switched on and R_Si_ is significantly lowered (Fig. S3), an applied voltage is distributed across all the circuit elements, with a voltage drop proportional to each element’s resistance (impedance). For a capacitor, the impedance (*Z* = *1/(2πfC)*) and the voltage drop (*V*_C_SI_) increase as the AC frequency (*f*) decreases. Therefore, we observe that a low AC frequency and a negative DC bias both prompt aid the HER as both effects enhance the voltage drop on the EDL capacitor. Owing to the same principle, a reduced electrolyte resistance (*R*_*s*_) improves the HER by taking a smaller voltage drop and redistributing it to other circuit elements, including the EDL capacitor next to Si. The experimental observations, i.e., the improvement of the MRLI with the decrease of the AC frequency and the increase of negative DC bias and Na_2_SO_4_ electrolyte conductivity (Fig. 2b–d), all agree with the theoretical circuit-model analysis, and both indicate that light-controlled electrochemical reaction is the dominating mechanism for bubble generation.

It should also be noted that a DC field alone can also generate microbubbles. However, we study the AC E-field due to its importance in understanding the working mechanism and practically employ this strategy for robust bubble creation, as we find that the Si surface may more easily degrade with DC or low-frequency AC fields. This can be attributed to the FTO layer partially exposed to the solution due to the voids/grain boundaries in the 200 nm *α*-Si:H (Fig. S3), in which case, the FTO competes with the reactions on the light-activated Si surface, generating bubbles and damaging the Si layer in the process. The use of an AC field, on the other hand, lowers the conductivity of the Si’s capacitive component, and hence the total resistance of the Si. While this also reduces the effective voltage applied to the EDL, the reactions are shifted more to the Si, with fewer reactions taking place near the FTO layer. Indeed, experimentally, at a 10 kHz AC field, we observe that the Si maintains its integrity. For the electrochemical formation of bubbles, we therefore choose an optimized AC-frequency and further add a negative DC bias to obtain the best MRLI value that preserves the surface performance. However, it should be noted that there isn’t a universal frequency at which Si becomes permanently stable, since its integrity can depend on factors like film thickness, deposition method, and surface treatment. For the specific silicon films used in our study, under the employed electric field conditions, we observed stable Si performance and achieved optimized efficiency in microbubble generation.

The understanding of the bubble formation mechanism assists us in rationally improving the efficiency of the electrochemical system by adding hydrogen peroxide and surfactants (CTAC) for a lower redox voltage and surface tension, respectively (Fig. 2f, SI Note 2). With all conditions optimized, it only requires a light illumination of ~0.1 W/cm^2^, corresponding to the level of sunlight, to create a microbubble (532 nm laser; electrolyte: 1.5 M Na_2_SO_4_, 1.25 mg/mL CTAC, 5% H_2_O_2_; *V*_pp_: 12 V at 1.5 kHz, square wave, *V*_bias_: −6 V) Even in PBS solutions, as low as ~0.22 W/cm^2^ is needed for bubble creation (532 nm light, *V*_pp_: 2 V at 1.5 kHz, *V*_bias_: −9 V). For biological applications, solutions such as PBS are recommended, without the addition of CTAC or H_2_O_2_ due to potential biocompatibility issues. Overall, this technique exhibits a much higher energy efficiency (>2–3 orders of magnitude) compared to other current bubble generation techniques listed in Table S2. This work, although using a similar setup to optoelectronic tweezers^28^, the mechanism is distinct, which generates bubbles and uses them for manipulation. In addition, our electrochemical bubble-based approach is fully compatible with high-ionic-strength solutions, overcoming the limitations of dielectrophoresis-based manipulation under such conditions.

### Fast, parallel, versatile microbubble patterning

**Fig. 3:**
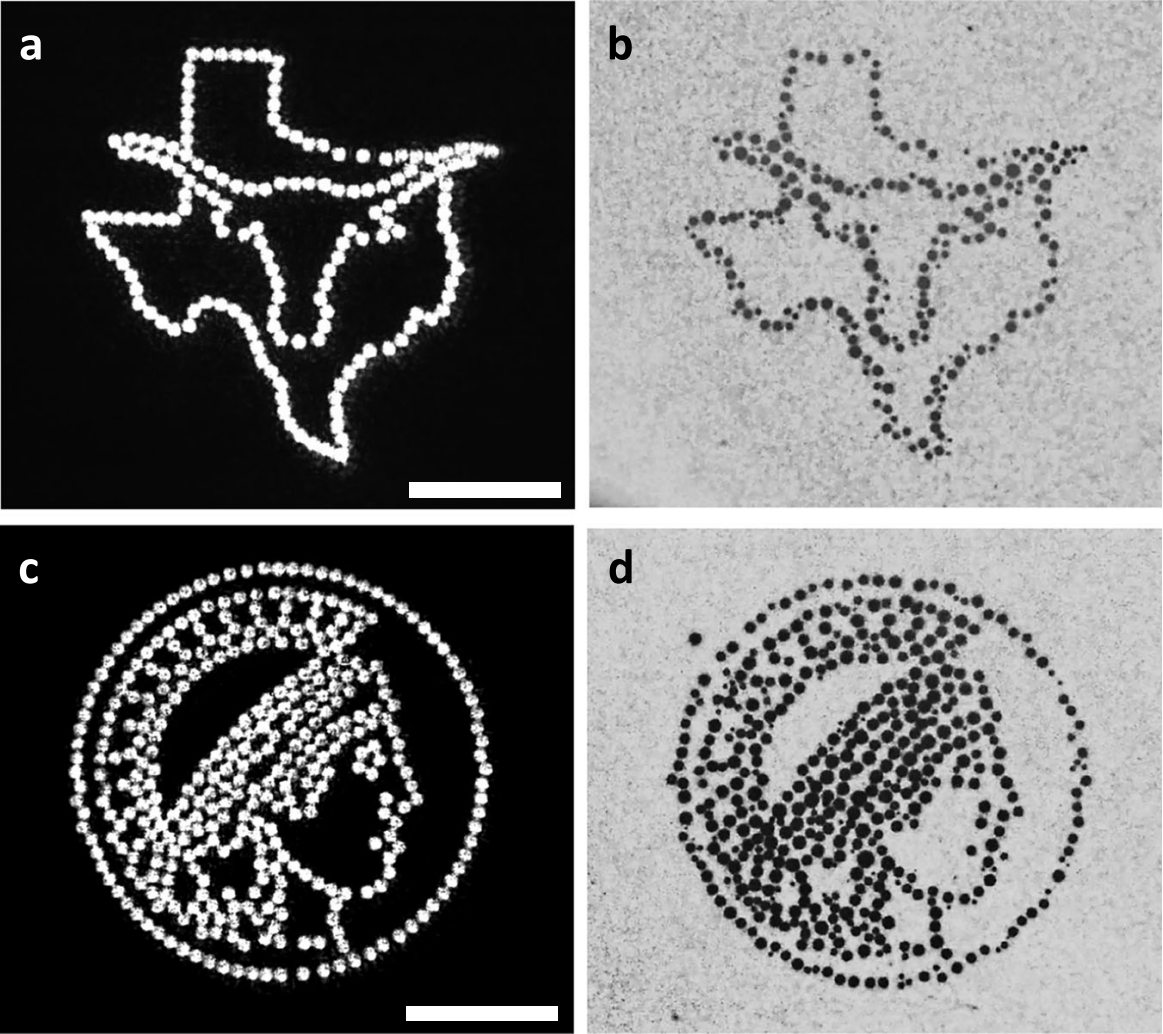
Demonstration of rapid, parallel, versatile microbubble patterning. **a** Laser profile and **b** corresponding bubble pattern of a Bevo superimposed on the map of Texas (Bevo is UT-Austin’s copyrighted logo). **c** Laser profile and corresponding **d** bubble pattern of the Max Planck Society’s logo. Scale bars: 100 μm.

The microbubble patterns can be rapidly refreshed to form dynamic movies. Demonstrations include a bubble-made longhorn image, the symbol of UT Austin, which is overlaid on the map of the state of Texas (Fig. 3a, b, Video S2), the logo of the Max Planck Society (Fig. 3c, d, Video S2), as well as animations, including a running horse (Video S3), a continuously expanding circle and fireworks (Video S4).

#### High-precision, bubble-actuated nanoparticle printing

**Fig. 4:**
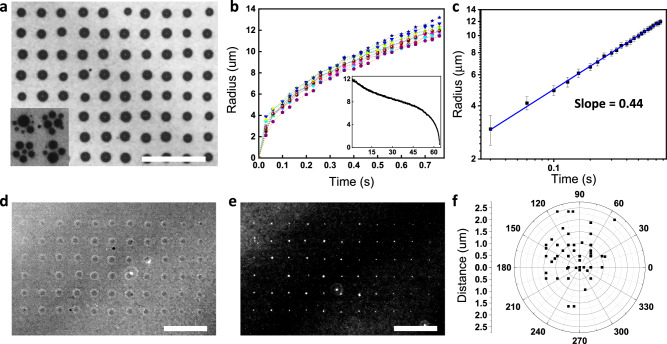
Role of nanoparticles in the generation of bubble patterns. **a** Simultaneous generation of microbubbles over a larger area, respectively, with nanoparticles in solution (inset without nanoparticles in solution). **b** A microbubble is generated rapidly, within sub-milliseconds (a few µm) to less than a second (~10 µm), and (inset) shrinks over tens of seconds. Results of multiple individual bubbles show consistent behaviors. **c** The size of the nanoparticle assembly can be controlled by the bubble size from a few µm to >100 µm. Formation of nanoparticle-assemblies in an array by **d** the generation of an array of microbubbles (shown as an optical micrograph) in a solution containing polystyrene nanospheres, followed by the **e** shrinkage of the microbubble arrays that result in ordered nanoparticle assemblies. **f** The location of the nanoparticle assembly is within 1.0 ± 0.6 μm relative to the center of the bubble position. Scale bars: 100 μm. Error bars: standard deviation (s.d.). Source data are provided as a Source Data file.

For capturing and assembling colloidal nanoparticles, it is crucial to control the surface state of the substrate and the concentration of the nanoparticle suspension. For instance, a native α-Si:H surface can only support truncated spherical hydrogen bubbles that results in coffee-ring particle patterns (Fig. S4) due to the hydrophobic nature of both hydrogen gas and α-Si:H^29,30^. Without nanoparticles in solution (or at a low concentration), multiple, instead of single, bubbles form under a light spot (Fig. 4a, inset and Video S5)^30^, which is detrimental to precise particle printing. The formation of single or multiple bubbles can be understood when considering the thermodynamic factors driving the bubble formation (SI Note 3). Nanoparticles are attracted to the bubble interface^25^, captured, and finally deposited at a single spot during its shrinkage (Fig. 4b, inset, SI Note 4).

The above observations, together with further considerations of surface charge and particle density (SI Note 5), indicate that surface modification is needed to enable the formation of single-spot particle aggregates. We first modify the *α*-Si:H surface with positive charges using an oxygen plasma followed by poly-diallyldimethylammonium chloride (PDDA) coating. The efforts generate single, size-controlled, and position-defined bubble patterns at the point of the incident light with a positional accuracy of 2.45 ± 1.08 µm (Fig. S5). The diameter of a bubble increases during the application of an E-field (Fig. 4b) (*V*_bias_: −5 V, *V*_pp_: 12 V at 1.5 kHz) with the power-law coefficient determined as 0.44, close to 0.5 for gas-diffusion controlled bubble growth (Fig. 4c and SI Note 6)^31,32^. The PDDA functionalization of Si further enables single-point contact of a bubble on the substrate; it also effectively helps in fixing the nanoparticles to the oppositely charged substrate (200-nm polystyrene nanospheres: 6–8 × 10^10^/ml; Na_2_SO_4_ to 0.66–0.75 M) (Fig. 4d, e and Video S6). In addition, the microbubbles shrink symmetrically during the process so that the nanoparticle clusters can be precisely localized to within 1.03 ± 0.61 μm of the microbubble center. This permits positioning to an order-of-magnitude smaller feature sizes compared to the size of the light pattern and bubbles (Fig. 4f).

The electrochemical microbubble platform can be used to reliably deposit a variety of inorganic and biological particles, ranging from 200 nm to several micrometers in size, onto the Si surface. The assemblies remain firmly attached, even when the surface is rinsed with DI water. The particle–particle and particle–surface adhesion can be attributed to electrostatic and van der Waals forces after bubble shrinkage (Fig. 4e). However, when a new microbubble is formed nearby, then the assembled particle cluster can be detached and repositioned, specifically, during bubble shrinkage. Microbubbles may thus not only be used to form stable assemblies, but also be used to reconfigure the particle organization.

#### Microbubbles for rapid programmable nanoparticle assembly

**Fig. 5:**
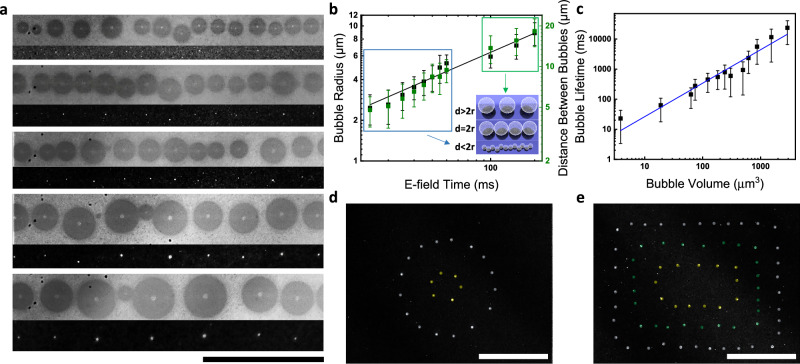
Demonstration of microbubble-driven nanoparticle cluster formation upon illumination of a large-area light pattern. **a** The interparticle distance of the deposits is controlled by the bubble size. Dependence of **b** (Black) bubble radius and (Green) separation of bubbles as a function of the E-field duration, and **c** bubble volume and bubble lifetime. Particle clusters formed by (**d**) circular and (**e**) rectangular light patterns (and the corresponding microbubbles). Simultaneously deposited particles (in the same color) are denoted in white, green, and yellow; enhanced images. Scale bars: 100 μm. Error bars: standard deviation (s.d.). Source data are provided as a Source Data file.

While small light spots can be used to create corresponding individual microbubbles with precise position control, simple large-area light patterns can also be applied to simultaneously generate many microbubbles for rapid, large-scale, programmable particle assembly (Fig. 5, Video S7). An average light intensity as low as 0.15 W/cm^2^ is sufficient to generate bubbles (*V*_bias_: −5 V, *V*_pp_: 12 V at 1.5 kHz, 0.75 M Na_2_SO_4_). A single bar of light can be used to obtain an evenly spaced chain of bubbles (Fig. 5a), where the spacing between bubbles can be controlled by the total E-field-application time (Fig. 5b). Depending on the sizes of the light pattern and bubbles, different arrangements of the patterns can be generated (inset of Fig. 5b, blue and green rectangles). When broadening the width of the light pattern, the bubbles first increase in size and then a second row of bubbles forms (Fig. S6). Further, the bubble size and hence the bubble lifetime (time for a bubble to collapse after growth) can be varied over three orders of magnitude from 23 to 24 s (Fig. 5c), with a corresponding increase of the number of deposited particles (Fig. S7). The nanoparticle assembly arises at the microbubble and by choosing appropriate parameters based on the above observations, we achieve programmable nanoparticle patterns consisting of evenly spaced spots along defined patterns (Fig. 5d, e).

#### Large-scale bubble printing

**Fig. 6:**
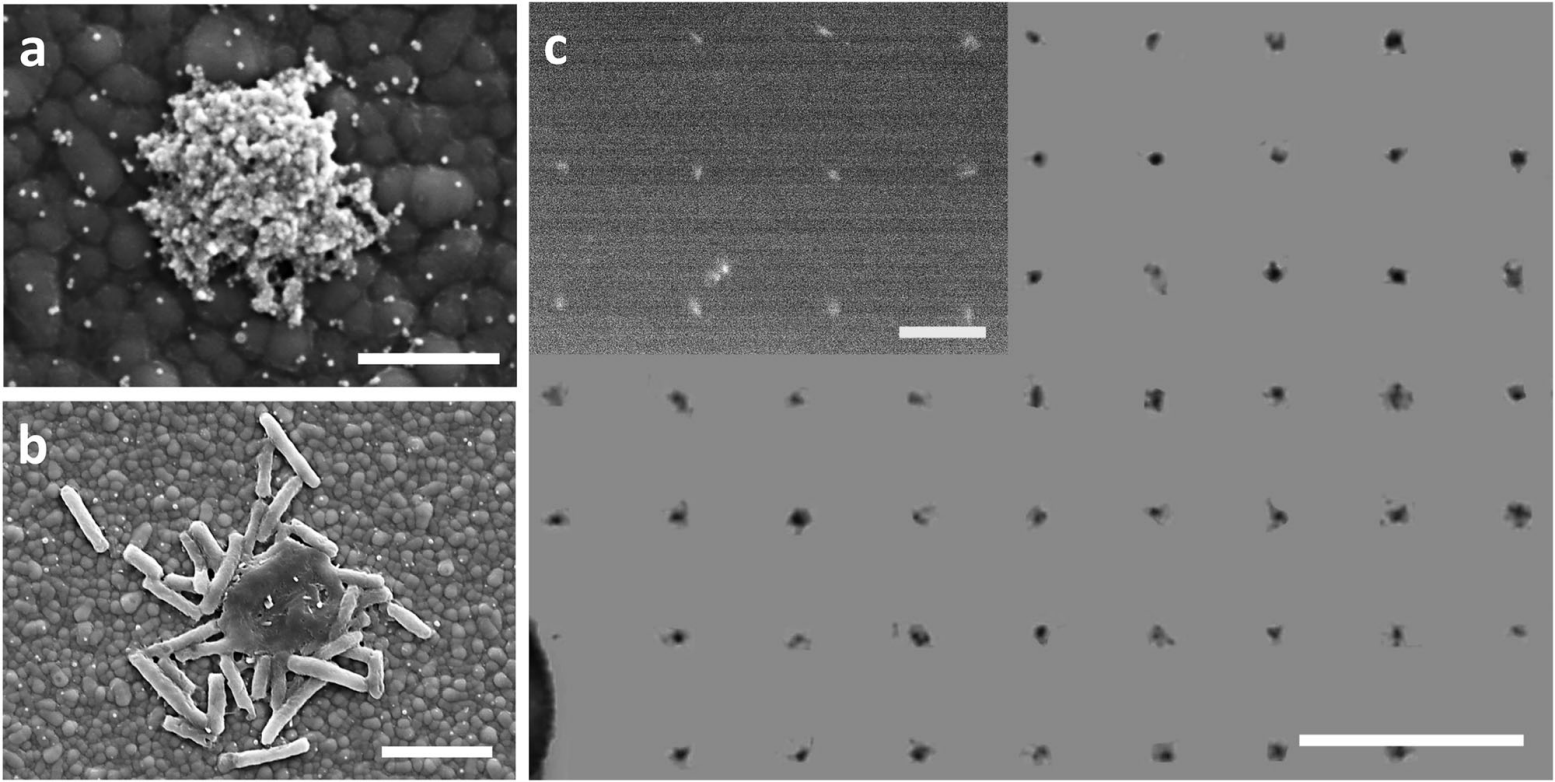
Microbubble patterning of diverse materials. Microbubble assembly of diverse structures ranging from **a** metallic silver nanoparticles (40 nm), **b** bacterial cells (E-coli) (1–2 mm), to **c** 200 nm extracellular vesicles from breast cells (enhanced image); original image in Fig. S13. Scale bars: **a** 1 μm, **b** 3 μm, **c** 50 μm, and 25 μm in inset.

Distinct from state-of-the-art printing techniques, opto-electrochemical bubble printing allows easy, massive, biocompatible parallel assemblies. We demonstrate the printing of Ag nanoparticles (40 nm), polystyrene spheres (200 nm), and live bacterial cells (1–2 µm) as well as assemblies of 200-nm extracellular vesicles in one exposure (Fig. 6), and a 2.56-mm-long nanosphere lattice in only 7 light exposures (Fig. S8). The aggregates are typically a few micrometers in size, and the deposition contrast can be further enhanced by optimizing the Si surface functionalization (Fig. S9a–c). These results suggest a facile approach to for instance enrich biologics in a complex solution by creating microbubbles and selectively depositing the desired species, assisted by surface-particle interactions. Bacterial cells, for example, assemble on positively charged silicon but redisperse into the solution when the surface is hydrophobic or negatively charged, while extracellular vesicles can be captured by hydrophobic surfaces^33^.

#### Microbubble platform for bioassays

**Fig. 7:**
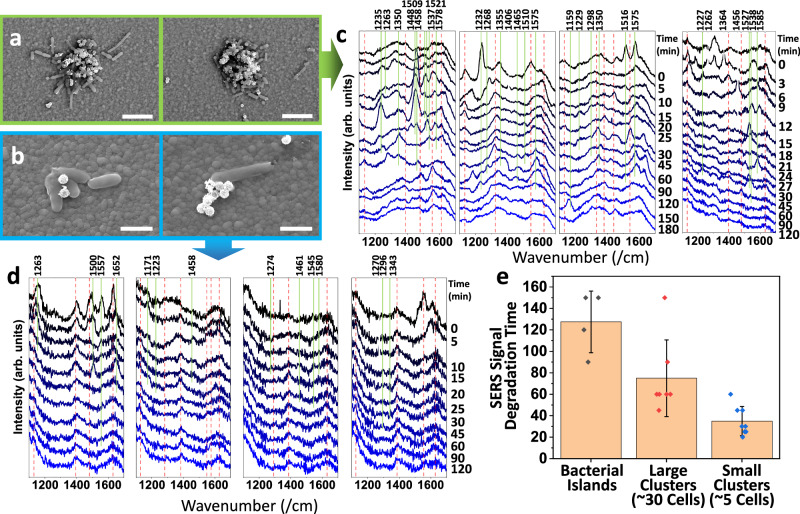
Co-assembly of nanosensor/cell hybrid arrays for metabolite profiling and drug response assay. Nanosensors: Ag-coated nanospheres; cells: Gram-positive B. Subtilis. Controlled cell counts in the hybrid arrays are determined by bubble size, ~98 µm for (**a**) and ~54 µm for (**b**). Continuous metabolite profiling up to 3 h in response to vancomycin for (**c**) large clusters in (**a**), and **d** small clusters in (**b**), exhibits (**e**) distinct diminishing time depending on average cell counts analyzed with Mann–Whitney U Test. Island-large clusters: *Z* value = −1.98; Large clusters-small clusters: *Z* value = −2.78; Islands-small clusters: *Z* value = −2.72. Raman signals in green and red for metabolites and the nanosphere sensors, respectively (**c**) **d** shows 4 representative measurement results. Additional data for (**c**, **d**) are available in Fig. S14. Scale bars: (**a**) 5 μm and (**b**) 2 μm. Error bars: Standard deviation (s.d.). Source data are provided as a Source Data file.

In addition, the rapid co-assembly of cells and nanosensors for continuous profiling of biomarkers can be achieved with microbubbles. As a proof of concept, we generate microbubbles in solution containing both bacterial cells and plasmonic nanosensors and co-assemble these in defined cell clusters, ranging from individual to tens of cells, on the substrate (Fig. 7a, b). The microbubble assembly exhibits excellent biocompatibility (>93%), as confirmed by standard trypan blue cell-staining of Chinese Hamster Ovary (CHO) cells after being exposed to microbubbles (see “Methods” section). Here, we focus on a bacteria–nanoparticle sensor array to highlight both the biological relevance and the unique strengths of our microbubble platform compared to the other technologies (Tables S3 and S4). The array enables the continuous monitoring of metabolite release from bacteria and provides biological insights into bacterial antibiotic resistance. Different cells can be assembled, such as *Escherichia coli* and *Bacillus(B.) Subtilis*, which remain highly active as shown by their strong, dynamic, and heterogenous metabolite release, without signs of degradation during the 2-h long continuous monitoring by surface-enhanced Raman scattering (SERS) (Fig. S10).

Here, we evaluate *B. Subtilis* in response to Vancomycin, an antibiotic drug commonly applied to Gram-positive bacteria. The SERS-active nanosensors are made of Ag-NPs synthesized on polystyrene (PS) nanospheres (Fig. S11, “Materials and Methods”), which are deposited along with *B. Subtilis* cells during the microbubble printing. We successfully detect the evolving metabolite signals from assembled arrays in a prescribed manner, as seen in the 4 representative SERS measurements in Fig. 7c, d. SERS reveals metabolites that are associated with end products of the purine cycle, including hypoxanthine, xanthine, uric acid, guanine, AMP and adenine, the metabolism of which is attributed to nutrient-deprived bacteria (Figs. 7c, d,S12 and S13; note that peaks associated with various metabolites shown in these figures are listed in Table S5, which provides Raman peak positions of purine derivatives identified in the literature)^34^.

Overall, we observe consistent strong heterogeneity of metabolites in terms of species and dynamics across cell assemblies ranging from a single cell to large aggregates with tens to hundreds of bacterial cells. The smaller the assembly size, the earlier the metabolite activity disappears in the presence of excess antibiotic drugs. Specifically, a small bacterial assembly with a few cells, e.g., 5 cells, remains active in releasing metabolites for ~35 min; the activity extends to ~75 min for tens of cells, e.g., 30, and ~120 min for bacterial islands or films with hundreds of cells (Fig. 7e). A detailed analysis is included in SI Note 7. The time-dependence of the drug response on cell-assembly size, discovered with this platform, could likely be attributed to the physical coverage and protections among cells with increased counts as well as their chemical communication^35^. Moreover, with a greater number of cells deposited in an assembly, the chance of including cells with antibiotic resistance increases, as well as the observed drug-response time. The microbubble technique is general and versatile and can thus be used to establish biosensing platforms for cell biology, drug screening, testing for precision therapeutics, and cell-cell communication studies.

### Summary

We introduce an opto-electrochemical technique that permits the room-temperature formation and patterning of microbubbles with defined size in a parallel and scalable fashion. The required low light intensity (~0.1 W/cm^2^), a few orders of magnitude less than what has been reported previously, triggers the electrolysis-driven bubble formation. The size of the microbubbles determines their lifetime, and the preparation of the substrate enables the formation of well-defined microbubbles. The use of nanoparticles in the suspension facilitates the growth of single bubbles, which guides the assembly of nanoparticle clusters onto a substrate. The nanoparticle aggregates can be positioned with an accuracy of ~1 µm, relative to the bubble center. The microbubble technique presented herein is general and can be used to quickly form precise assemblies from a range of structures and materials with ease, including polymeric or metallic nanoparticles, extracellular vesicles, and live biological cells. For demonstration, we pattern arrays of plasmonic-active nanosensor-cell hybrids and record the dynamic metabolite release from bacterial cells of different assembled sizes for several hours. The biocompatible microbubble assembly process also permits the dynamic evaluation of controlled microbial clusters in response to antibiotics, which facilitates research into cell signaling, metabolism, and drug development, including antibiotics. The microbubble-based assembly offers several key advantages for cell manipulation and patterning. It enables one-step trapping and attachment of cells to a substrate, without requiring the complex microfabrication typically needed for microfluidic devices, valve-based platforms, or physical traps. Additionally, the bubble-based system can capture a wide variety of particles across a broad range of sizes and properties, making it particularly effective for assembling cell/nanosensor hybrids. A detailed comparison with other state-of-the-art techniques, such as microfluidic processing^36,37^ and active cell trapping^34,36,38^, is presented in Table S3 of SI. We expect that the fast, massively parallel small-particle assembly method presented herein, will enable further applications in nanomanufacturing^39^, nanophotonic patterning^40^, nanorobotics^41–43^, biosensing^44,45^, single-cell biology^46^, and hybrid device fabrication^44^. In particular, this research overcomes longstanding challenges in obtaining microbubble patterns with both high precision and high throughput. These unique capabilities make for general soft microtools and pave the way for cell-sensor-stimulus platforms for cell biology, drug screening, cell-cell communication studies and precision therapeutics.

## Methods

### Experimental setup

In a typical experiment, a 100–250 μm-thick PDMS well (1.5 mm in diameter) is placed on the α-Si:H surface that has been cleaned by oxygen plasma or UV-ozone treatment. A Na_2_SO_4_ solution (0.18 μL) is mixed with the nanoparticles that are to be deposited and then dispersed into the well. The well is sealed with a FTO glass, which serves as the counter-electrode. The FTO on the α-Si:H layer serves as the working electrode. An electric voltage is applied between the working and counter electrodes. The light-illuminated areas become conducting and here the bubbles are formed by electrolysis.

### Characterization of the minimally required laser intensity

A single laser beam with a diameter of 7.22 μm is used for the characterization of the bubble formation, measured by the 10/90 knife-edge method (Fig. S12). Prior to the bubble generation, the surface of the silicon was gently cleaned using DI water and IPA. Unless stated otherwise, a 0.1 M Na_2_SO_4_ solution, −5 V DC / 12 Vpp at 1.5 kHz AC E-field was applied for ~225 ms using a 500 μm PDMS well for characterization purposes. The electrolyte solutions were prepared before use from a 1.5 M Na_2_SO_4_ stock solution. When testing the effects of H_2_O_2_ and CTAC these molecules were added to achieve a final concentration of 5% and 1.25 mg/mL, respectively.

### Microbubble patterning and particle deposition

For bubble-driven nanoparticle assembly, the substrate surface is first cleaned with DI water and IPA. This is followed by a treatment with oxygen plasma for 1 min at 50 sccm, 50 W power, or with a UVO cleaner for 30 min. A 4% PDDA solution is then applied to the surface for 3 m to positively charge the surface and increase the hydrophilicity, after which the surface is washed with DI water and gently dried using a flow of nitrogen gas. A 0.66–0.8 M Na_2_SO_4_ solution containing 200 nm polystyrene nanoparticles is added to the PDMS well applied on the surface. A light pattern is projected via a digital mirror array controlled by a custom-made python program using a continuous 532 nm laser (DJ532-40, Thorlabs). An E-field of −5 V DC and 12 *V*_pp_ AC voltage at 1.5 kHz is applied between the working electrode underneath the α-Si:H surface and the counter FTO electrode. Microbubble patterns were formed with 15–50 ms AC bursts, while the bubble-assisted nanoparticle assembly were formed with bursts lasting 225–750 ms.

### CHO cell culturing and viability testing for biocompatibility determination

CHO cells were cultured in RPMI medium supplemented with 10% fetal bovine serum and 1% penicillin-streptomycin and maintained in an incubator at a 37 °C, 5% CO_2_ environment. After culturing, the cells were detached from the culturing flask and diluted to achieve a concentration of 10^6^ cells/mL. A cell solution was applied to a PDMS well on top of PDDA-treated α-Si:H, and bubbles were formed throughout the sample surface following standard microbubble patterning procedures, as described above. Once the bubbles are no longer present, trypan blue was added to the solution. The viability of the cells was determined by checking whether the cells are stained with the dye after waiting 3–5 min. To ensure accurate assessment, only cells that in direct contact with microbubbles were considered, and over 360 cells were analyzed, showing a livability of >93%.

### Bacterial microbubble assembly and characterization

For bubble-based live *E. coli* deposition, the *E. coli* was washed with DI water for 3 times and concentrated to 4.77 × 10^7^ cells/mL. The concentrated solution was mixed with a 1 M Na_2_SO_4_ stock solution 1:1 to facilitate bubble growth, resulting in a 10×, 0.5 M Na_2_SO_4_ *E-coli* solution. For SEM imaging, the original *E. coli* was fixed via washing with a 7.4 PBS solution 2 times and submersing in a 1:3 acetic acid:EtOH solution for 10 min^47^. Then, the solution was washed by PBS solution twice and finally washed by DI water and concentrated to obtain a 20× higher *E. coli* cell density. The concentrated solution was mixed with a 1 M Na_2_SO_4_ stock solution 1:1 to facilitate bubble growth, resulting in a 10×, 0.5 M Na_2_SO_4_ *E. coli* solution. After depositing the *E. coli* with bubbles, the solution was gently exchanged with DI water 10 times to suppress random deposition of *E. coli* on the surface, then dried. Before SEM imaging, a thin layer of Au/Pd was deposited to improve the imaging of the samples.

### Fabrication of SERS-active Ag-on-PS nanospheres, microbubble co-assembly of SERS-nanosensor/*B. Subtilis*-cell hybrids and dynamic profiling of metabolites with SERS

Dense and uniform Ag nanoparticles were synthesized on commercial 500 nm PS nanospheres (Alfa Aesar, 42714) for SERS nanosensing by using a previously reported method with minor modification^45,48,49^. First, 1.5 ml of 0.06 M NH₄OH solution was injected into a centrifuge tube, followed by 0.75 ml of 0.06 M AgNO₃ to obtain a stable Ammonia-Silver Ion Complex (solution A). Then, 98 µl of the PS spheres solution (2.5 wt%) was added to solution A and shaken for thorough mixing. An additional 0.75 ml of 0.06 M AgNO₃ was gradually added to solution A. The silver oxide core would form with the solution injection, turning the solution color from white to light yellow (solution B), which was shaken for another 45 min for ripening. Afterward, 2 ml of solution B was removed, leaving 1 ml in the tube to allow further growth of the silver nanoparticles. Then, 10 ml of PVP (2 × 10^−5 ^M in ethanol, M.W. 40,000) was added to the tube as a reductant and stabilizer. The tube was then placed in a 70 °C oven for 7 h to produce dense silver particles with a diameter of around 50 nm on the PS nanospheres. To remove the remaining PVP for clear and sensitive SERS detection, the particles were stored in a 0.1 M KCl solution for a day and treated with a 0.025 M NaBH_4_ solution for 10 min and then washed two times with DI water before use. *B. Subtilis* were washed for 5 times in DI water and concentrated before use (4.3 × 10^8^ cells/mL). The particles, cells, and a 1 M Na_2_SO_4_ solution were mixed with a volume ratio of 1:1:1 for microbubble deposition. After deposition, the solution was removed and replaced with a Vancomycin solution (8 μg/mL) for SERS testing.

### Generation of MDA-MB-231-PalmGRET stable cell lines

To generate the MDA-MB-231-PalmGRET stable cell line, pLenti CMV Puro DEST plasmids encoding PalmGRET (Plasmid #158221; Addgene) were packaged into lentiviruses, then transduced into MDA-MB-231cells. Next, puromycin selection (10 μg/ml, Invitrogen) was conducted to eliminate non-transduced cells. Additional selection using fluorescence-activated cell sorting was performed to screen for cells that highly expressed PalmGRET.

### Collection of MDA-MB-231-PalmGRET extracellular vesicles

Cells were grown in T225 flasks (Falcon) until they reached full confluency. Next, cells were cultured in serum free medium (Corning) for 22–24 h. The medium was collected and centrifuged at 800 × *g* for 5 min followed by a centrifugation step at 2500 × *g* for 15 min to discard cellular debris and large particles. The supernatant was collected and continuously ultracentrifuged at 100,000 × *g* and 4 °C for 2 h. The EV pellets were suspended in PBS.

## Supplementary information

Supplementary Information

Description of Additional Supplementary Files

Video S1

Video S2

Video S3

Video S4

Video S5

Video S6

Video S7

Transparent Peer Review file

## Source data

Source Data

## Data Availability

The authors declare that the main data supporting the findings of this study are available within this paper and its Supplementary Information Figures. Source data are provided with this paper.
