# Supplementary material for: Massively parallel microbubble nano-assembly
Source: Nat Commun. 2025 Jul 22;16:6743. doi: 10.1038/s41467-025-62070-9 (PMC12284250; doi:10.1038/s41467-025-62070-9)
Supplement: Supplementary file 1 — Supplementary Information [file 41467_2025_62070_MOESM1_ESM.pdf]

Supplementary Information for

Massively Parallel Microbubble Nano-Assembly

Hyungmok Joh[1], Bin Lian[1], Shaw-iong Hsueh[2], Zhichao Ma[3], Keng-Jung Lee,[4] Si-yang Zheng,[4,5] Peer Fischer[6, 7, 8, 9*], and Donglei Emma Fan[1, 2, 10*]

[1] Materials Science and Engineering Program, Texas Materials Institute, The University of Texas at Austin
[2] Chandra Family Department of Electrical and Computer Engineering, The University of Texas at Austin

[3] Current address: School of Biomedical Engineering and Institute of Medical Robotics, Shanghai Jiao Tong University, China

[4] Biomedical Engineering, Carnegie Mellon University, Pittsburgh, PA 15213, United States

[5] Electrical & Computer Engineering, Carnegie Mellon University, Pittsburgh, PA 15213, United States

[6] Max Planck Institute for Medical Research, Jahnstrasse 29, 69120 Heidelberg, Germany

[7] Institute for Molecular Systems Engineering and Advanced Materials, Heidelberg University, INF 255, 69120 Heidelberg, Germany

[8] Center for Nanomedicine, Institute for Basic Science (IBS), Seoul 03722, Republic of Korea

[9] Department of Nano Biomedical Engineering (NanoBME), Advanced Science Institute, Yonsei University, Seoul, 03722, Republic of Korea

[10] Walker Department of Mechanical Engineering, University of Texas at Austin, Austin, TX 78712, USA

Supplementary Information Table of Content:

Supplementary Figures:

1. Supplementary Figure S1: Voltage-current characteristics of α-Si:H sample

2. Supplementary Figure S2: Simulation of heat generation

3. Supplementary Figure S3. Photocurrent measurement and cross-sectional SEM of α-Si:H sample

4. Supplementary Figure S4: Coffee ring patterns of dried particles for a hydrophobic surface

5. Supplementary Figure S5: Accuracy of bubble position relative to laser center

6. Supplementary Figure S6: Location of bubble position as a function of width of illuminated area

7. Supplementary Figure S7: Number of particles as a function of bubble volume

8. Supplementary Figure S8: Demonstration of large ordered arrays of nanoparticles

9. Supplementary Figure S9: Polystyrene and silver deposits as a function of surface charge

10. Supplementary Figure S10: SERS of PS-Ag-bacteria system and metabolite spectra

11. Supplementary Figure S11: SERS spectra of Ag on PS

12. Supplementary Figure S12: Determination of laser beam diameter

13. Supplementary Figure S13: Large-scale deposition of extracellular vesicles

14. Supplementary Figure S14: SERS metabolite spectra for assemblies made of different bubble sizes

15. Supplementary Figure S15: Measurement of bubble radius over time

16. Supplementary Figure S16: SERS metabolite spectra in response to Vancomycin

Supplementary Tables:

17. Supplementary Table S1. State-of-the-art bubble-based technologies

18. Supplementary Table S2. Comparison of Energy consumption of various current bubble-generation techniques

19. Supplementary Table S3. State-of-the-art individual cell manipulation and assembly methods

20. Supplementary Table S4. State-of-the-art antibiotic drug screening methods

21. Supplementary Table S5. Raman peak positions of purine derivatives for identification

Supplementary Notes:

22. Supplementary Note 1: Heating and simulation and laser heating

23. Supplementary Note 2: Effect of H_2O_2 and CTAC

24. Supplementary Note 3: Considerations on the formation of single vs multiple bubbles

25. Supplementary Note 4: Shrinkage of the H_2 bubbles

26. Supplementary Note 5: Consideration of the surface state and particle density

27. Supplementary Note 6: Gas-diffusion controlled process of microbubble growth

28. Supplementary Note 7: Measurement of *B. Subtilis* metabolites using SERS

1. Supplementary Figure S1: Voltage-current characteristics of α-Si:H sample

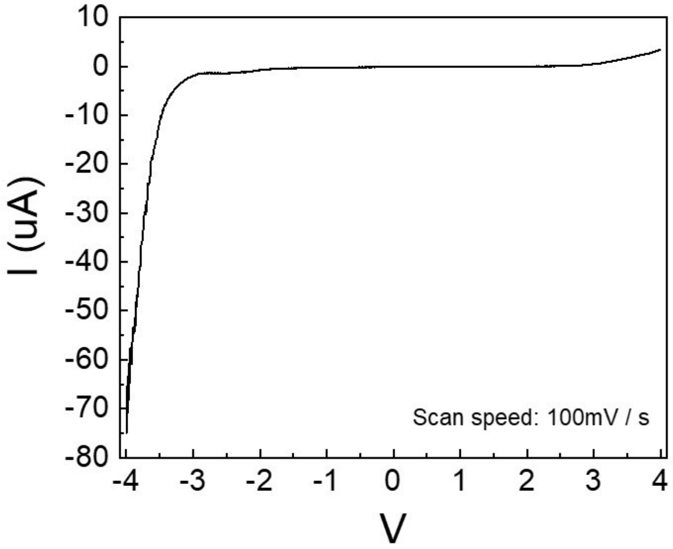

Figure S1. Voltage sweep from +4 V to -4 V of the α-Si:H sample used in this work.

2. Supplementary Figure S2: Simulation of heat generation

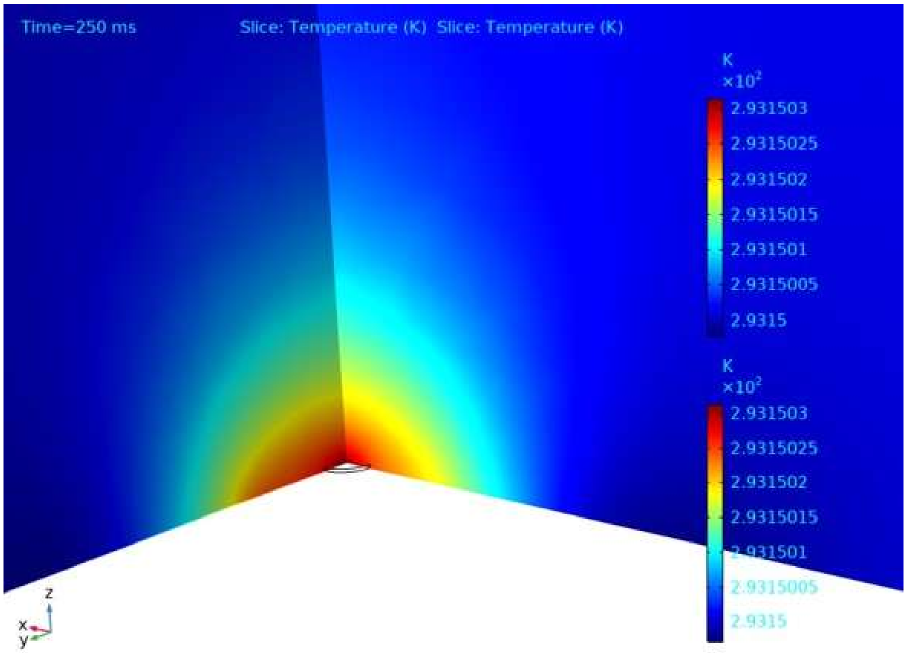

Figure S2. Simulation results of heat generated by a focused 532 nm laser with an intensity of 100 mW/cm^2. The initial temperature is set at 293 K, resulting in a temperature increase of ~0.15 K.

3. Supplementary Figure S3. Photocurrent measurement and cross-sectional SEM of α-Si:H sample

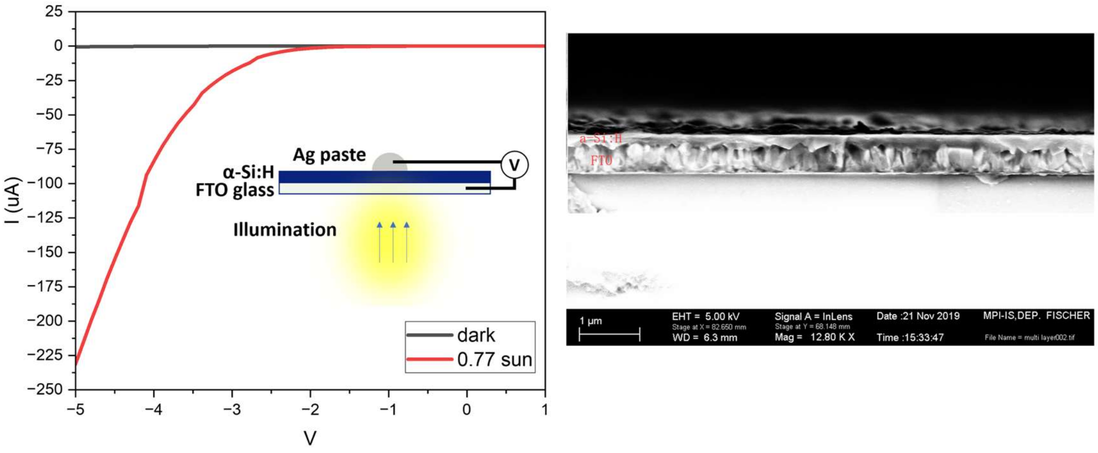

Figure S3. (left) Photocurrent and (right) cross-sectional SEM measurement results of an α-Si:H sample.

4. Supplementary Figure S4: Coffee ring patterns of dried particles for a hydrophobic surface

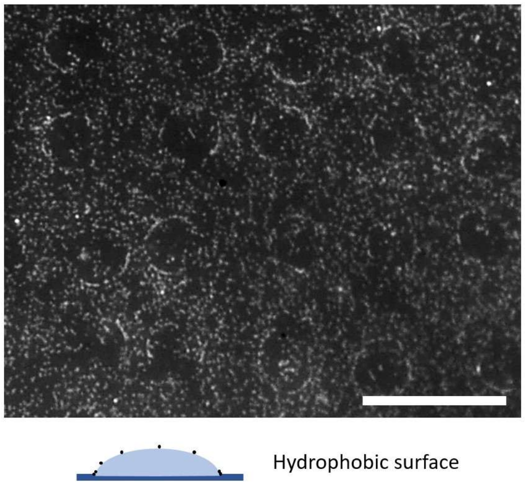

Figure S4. Coffee ring patterns form when the surface is hydrophobic. Image has been enhanced by adjusting the contrast / brightness / sharpness to show more clearly the deposited particles. Scale bar: 50 μm.

5. Supplementary Figure S5: Accuracy of bubble position relative to laser center

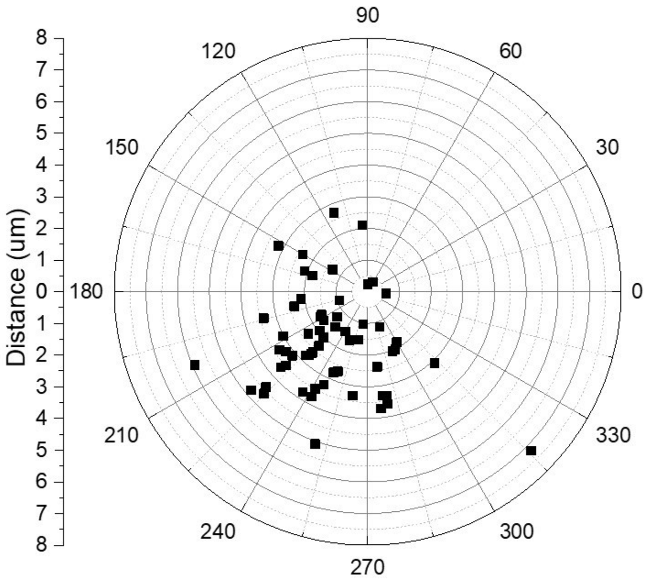

Figure S5. The center of the bubble position is within 2.45 ± 1.08 µm relative to the center of the laser spot.

6. Supplementary Figure S6: Location of bubble position as a function of width of illuminated area

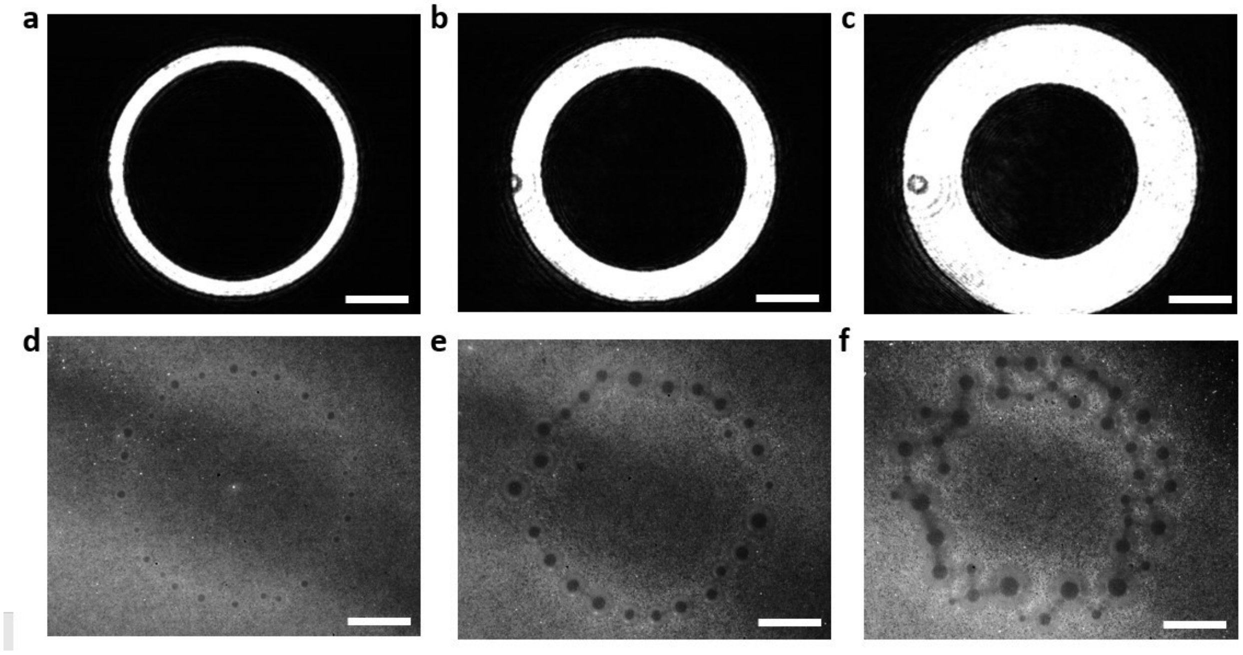

Figure S6. Laser patterns used for line thickness vs bubble pattern determination: a) 8 µm, b) 16 µm and c) 32 µm, and their corresponding bubble patterns. d-f) Scale bars: 50 µm.

7. Supplementary Figure S7: Number of particles as a function of bubble volume

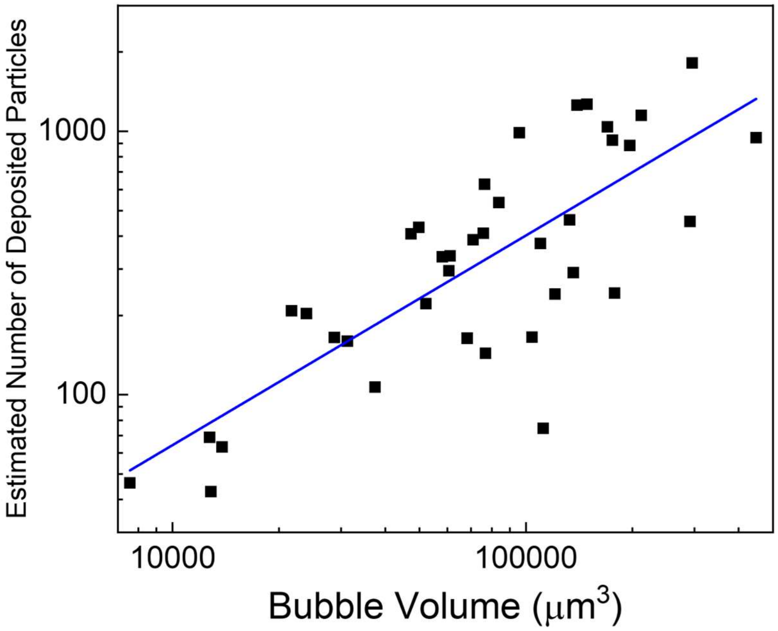

Figure S7. Experimental analysis shows the increase of particle deposition with the increase of bubble size.

8. Supplementary Figure S8: Demonstration of large ordered arrays of nanoparticles

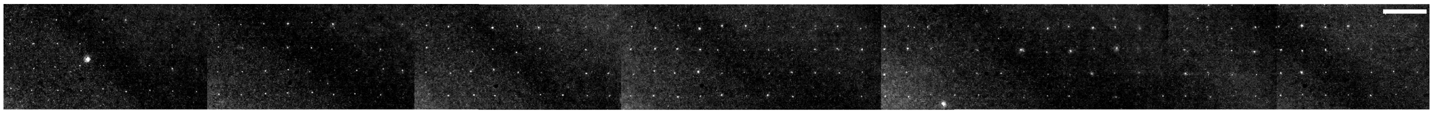

Figure S8. Bubble-assisted large-scale assembling of ordered arrays of nanospheres (total length: 2.56mm). Scale bar: 100 μm.

9. Supplementary Figure S9: Polystyrene and silver deposits as a function of surface charge

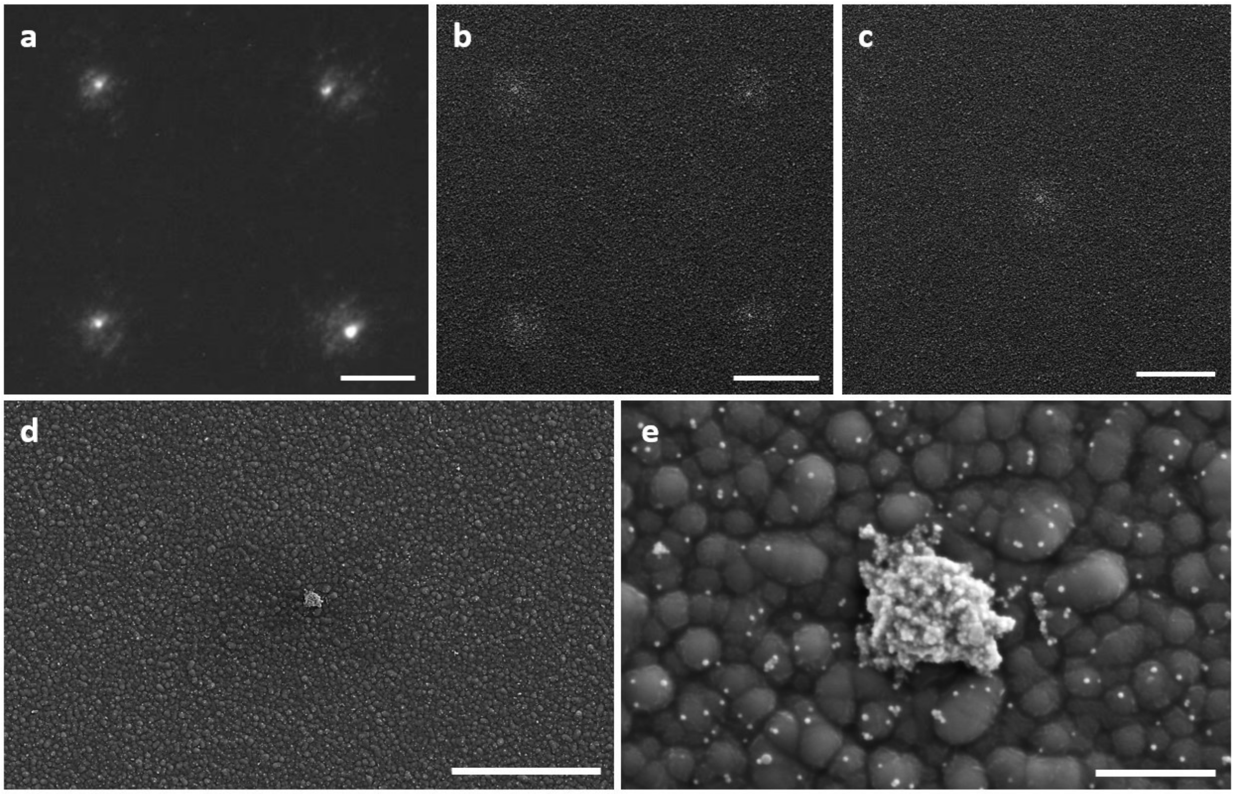

Figure S9. a) Fluorescent and b-c) SEM images of 2 x 2 PS sphere deposits on a negatively charged surface. Scale bars: 25 µm. d-e) SEM of a single Ag nanoparticle assembly on positively charged surface. Scale bars: 10 µm, 1 µm.

10. Supplementary Figure S10: SERS of PS-Ag-bacteria system and metabolite spectra

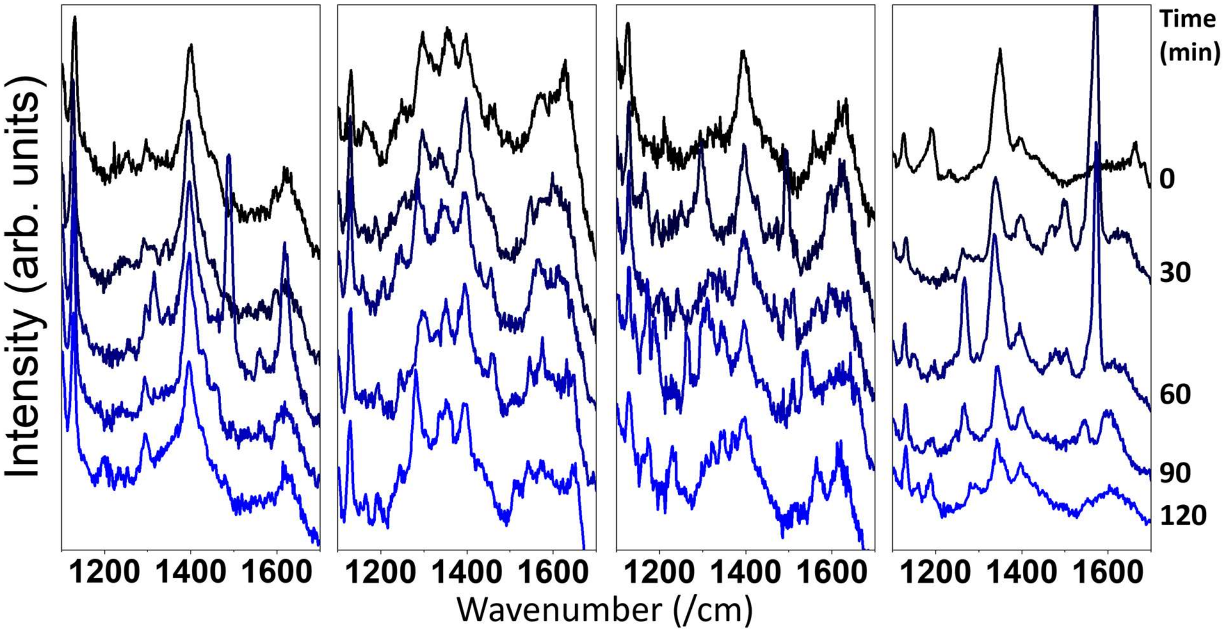

Figure S10. SERS of co-deposited PS-Ag spheres / B. subtilis cells in DI water. The metabolite release remains strong throughout the 2-hour SERS measurement.

11. Supplementary Figure S11: SERS spectra of Ag on PS

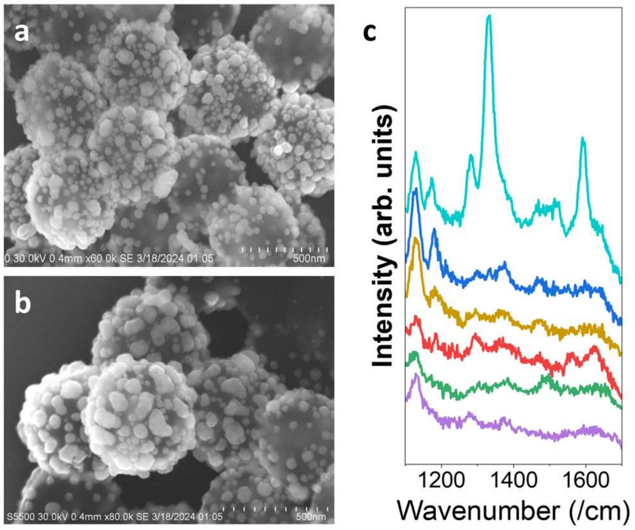

Figure S11 a-b) SEM and c) typical SERS spectra of Ag nanoparticles synthesized on PS spheres. Each colored curve in c) is measured from a different Ag-on-PS sphere aggregate.

12. Supplementary Figure S12: Determination of laser beam diameter

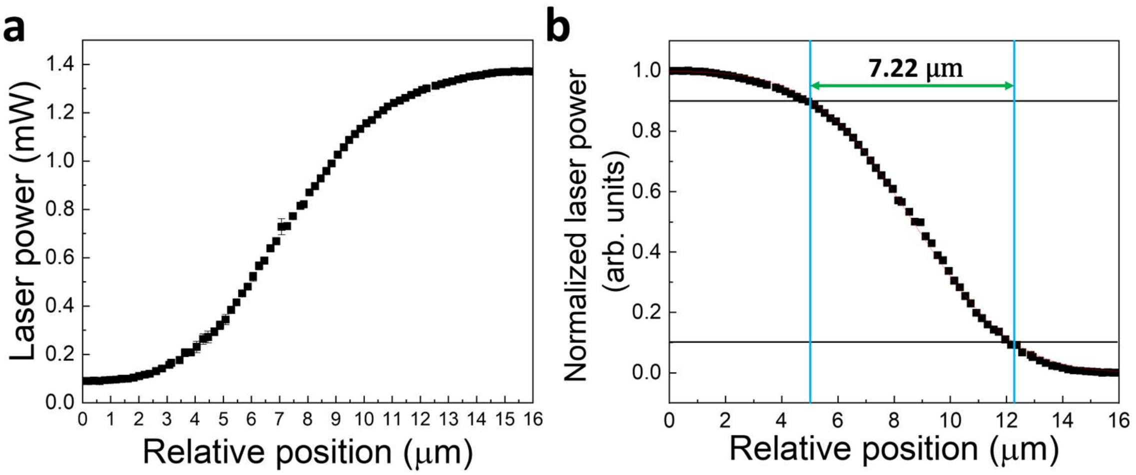

Figure S12. a) Original and b) normalized laser power vs relative position used for laser beam width determination with the 10/90 knife edge method.

13. Supplementary Figure S13: Large-scale deposition of extracellular vesicles

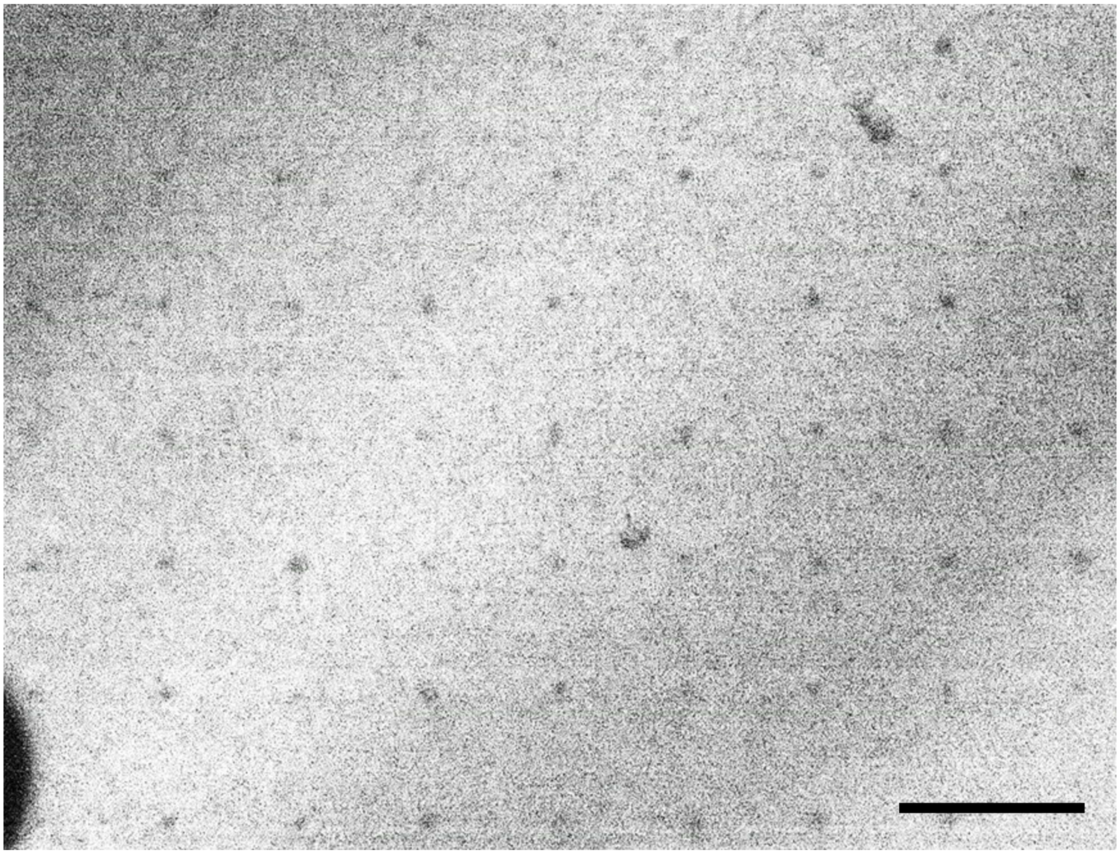

Figure S13. Original image of large-scale deposition of extracellular vesicles. Scale bar: 100 µm.

14. Supplementary Figure S14: SERS metabolite spectra for assemblies made of different bubble sizes

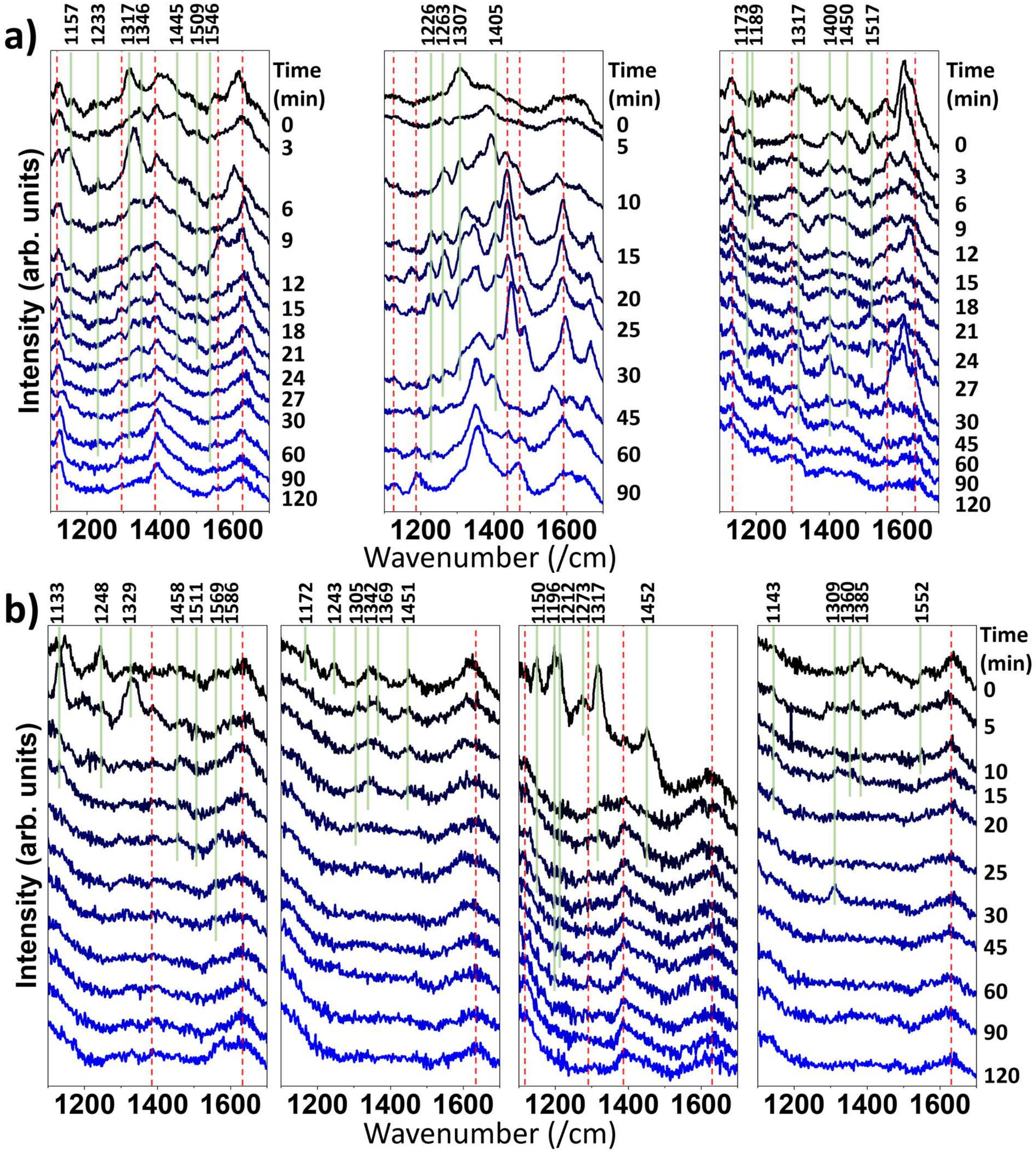

Figure S14. Additional metabolite detection of PS-Ag sphere/cell assemblies deposited by a) large bubbles of ~98 µm and b) small bubbles of ~54 µm in diameter.

15. Supplementary Figure S15: Measurement of bubble radius over time

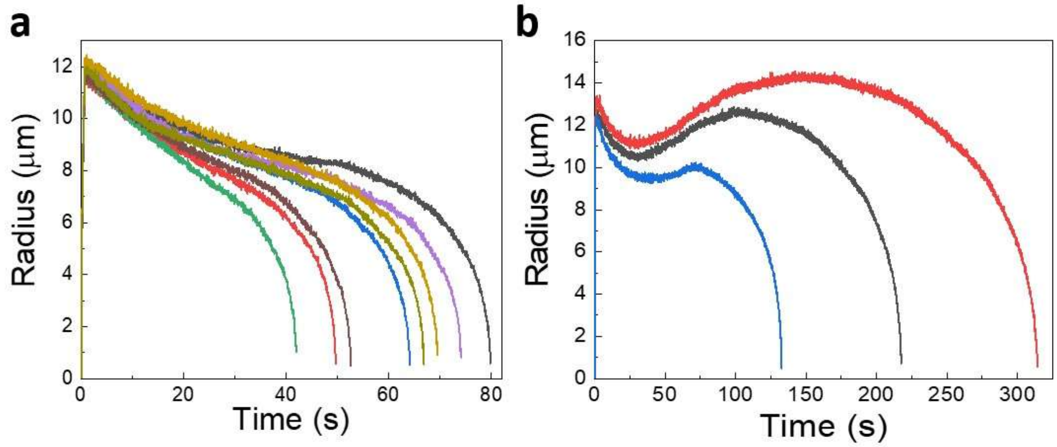

Figure S15. Analysis of bubble radius over time during bubble generation and collapse. a) While most of the bubbles shrank similarly over time, b) some bubbles showed a secondary expansion phase before shrinking again. Colored curves in a) and b) show the radius versus time of different bubbles.

16. Supplementary Figure S16: SERS metabolite spectra in response to Vancomycin

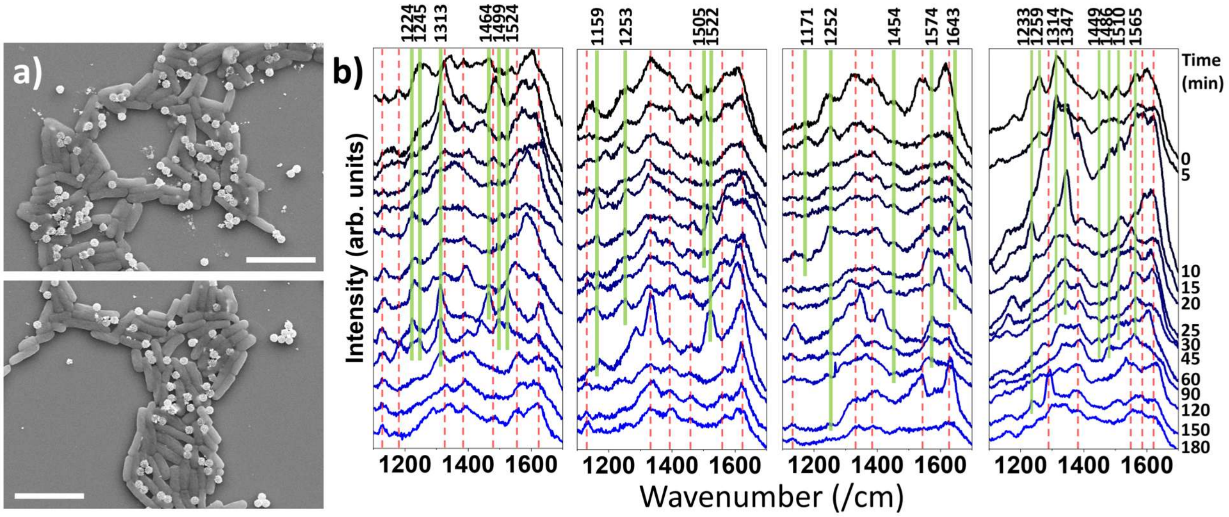

Figure S16. a) SEM of PS-Ag sphere / bacterial cell assembled island formed by solution drying immediately followed by antibiotic treatment and b) SERS of metabolites in response to antibiotic Vancomycin. Scale bars: 5 µm.

17. Supplementary Table S1. State-of-the-art bubble-based technologies [1–13]

Bubble Generation Mechanism	Application	Limitations	Reference
Template-based Bubble Trapping	Chemical Mixer	Complicated/multi-step fabrication processes<br>Only usable for each specific use case as templates cannot be modified<br>May induce clogging if bubble size is not controlled	1, 2
	Chemical Switch		3
	Drug Delivery		4
Opto-Thermal Bubble Generation	Actuators	Specialized substrates such as gold nano-islands may be needed<br>High temperature makes it difficult to work with biological samples<br>Significant energy input needed, limiting the method to serial processes	5
			6
	Particle Trapping and Printing		7, 8
Mechanical Agitation	Cell Sorting	Size distribution of bubbles can vary	9
Electrolysis on Pixelated CMOS chips	Acoustic Holograms	Complicated/multi-step fabrication processes<br>Size of bubbles vary and cannot be precisely controlled	10
Large Scale, Light-modulated Electrolysis		Precise control of bubble location not realized	11
Photocatalytic Reactions	Nano/Microrobot Propulsion	Created on individual particles that propel<br>Difficult to control and use<br>Often requires high light energy	12, 13

18. Supplementary Table S2. Comparison of Energy consumption of various current bubble-generation techniques[14–18]

Microbubble formation mechanism	Bubble size used for energy estimation	Estimated required energy	Spatial and Number control	ref
Filamentation-based Femtosecond Laser pulses	20 µm	100uJ x 500 pulses<br>= 50 mJ = 6.95 x 10E-4 Wh	Location determined by laser beam but multiple bubble formation which cannot be controlled	14
Optothermal utilizing plasmonic substrates	15 µm	E-06 – E-07 Wh by estimation	Location determined by laser spot, single bubble formation	15
Carbon Nanotube heating	~12 µm (estimated)	Value given by paper = 5.9E-7 Wh	Single bubble formation, but location determined by resistive element which cannot be controlled	16
Laser-induced breakdown	10 µm	Value given by paper = 2.8E-7 Wh	Location determined by laser spot, single bubble formation	17
Microplasma generated by single pulsed discharge in water	88 µm	Value given by paper = 1.5E-7 Wh	Controllable size and location, but the location is fixed to the tip and not on the surface	18
**Opto-electrochemical bubbles**	**15 µm**	**Estimated power (upper limit) ~ 6E-10 Wh**	**Fully dynamic spatial control with single bubbles made at desired locations**	This work

19. Supplementary Table S3. State-of-the-art individual cell manipulation and assembly methods [19–32]

Technology / technique	Advantages	Disadvantages	ref
**Droplet-based single-cell sorting (Microfluidics)**	- No need for sophisticated instruments<br>- Can obtain a community of substantially higher diversity and evenness after culturing<br>- Encapsulating individual cells in tiny droplets provides a protective environment for cells to grow without competition from others<br>- Thousands of cells can be cultured in parallel	- Complex fabrication processes<br>- Unintentional droplet merging of splitting may affect sorting accuracy<br>- May result in empty droplets and droplets containing more than one cell<br>- Ensuring cell viability during encapsulation may be challenging<br>- Shear stress and long processing times may cause cell death<br>- Difficult to study cell behavior simultaneously	19, 20
**Dilution of cell culture and spreading onto a substrate with microchambers**	- Can make multiple single-cell samples at once<br>- Simple, does not need any complicated setups for the fabrication of single-cell microchambers	- Most microchambers (7/8) are either empty or have more than one cell	21
**Microchannel/fluidic based platforms (Valve-based)**	- A valve-based microfluidic device allows single-cell capture, lysis and reverse transcription, etc. all on one chip<br>- Enables multi-step chemical reactions under different conditions (temperature) or with different reagents by separating the reactions in isolated chambers<br>- Low reagent consumption	- Complex fabrication requirements<br>- The need for valves for control	22
**Microchannel/fluidic based platforms (Pressure-driven)**	- No valve required; liquid flow controlled by air pressure with high accuracy	- Requires a pressure source, such as a gas pump or cylinder, adding complexity and cost to the setup<br>- Dynamic control of pressure may be tricky due to decrease in pressure as fluid flows through the system<br>- Complex fabrication processes	23, 24
**Cell picking by pipette**	- Cells are intact<br>- Highly selective	- Slow<br>- Highly dependent on user skill	25, 26
**Plate-based methods**	- Can generate full-length transcripts that carry ample information to allow detection of genes that are not highly expressed and other rare transcripts	- Single cells need to be sorted into each microchamber<br>- limited by plate size	27
**MACS (Magnetic-Activated Cell Sorting)**	- Based on antibodies, enzymes, lectins, or streptavidin conjugated to magnetic beads to bind specific proteins on the target cells<br>- High specificity<br>- Cost effective	- Non-specific cell capture<br>- Requires magnetic particle labeling	28
**Single Cell capture via dielectrophoresis**	- Cells are intact<br>- Single cell capture is available	- Requires microfabrication of electrodes<br>- Does not attach bacterial cells on the surface for measurements once E-field is removed, while continuous application of E-fields can alter cells' physiology	29
**Microstructure-based methods (traps)**	- Can be designed to accommodate different cells sizes and types<br>- Single cell capture is available	- Trap clogging<br>- Complex fabrication requirements, especially for those requiring smaller feature sizes<br>- Requires precise flow control	30
**Acoustic field-based traps**	- Large-scale single-cell manipulation<br>- Label-free approach, ensuring high biocompatibility<br>- Effective across all cell sizes	- Microfabrication of electrodes are needed for acoustic wave generation<br>- Trapping particles with different sizes simultaneously can be challenging due to different optimal frequency<br>- No intrinsic mechanism for attaching cells directly onto the surface<br>- Patterning requires the acoustic waves applied continuously	31, 32

20. Supplementary Table S4. State-of-the-art antibiotic drug screening methods[33–47]

Technique	Working Mechanism	Cell counts controlled (a few to many)	Dynamic, continuous Monitoring	Sensing target/Method	Equipment Complexity	Required time	Special Requirement	References
Optical microscopic Imaging	Microscopic imaging to quantify morphological changes induced by antibiotic drugs in microfluidics on single cells	No	**Yes**	Imaging of cell morphology	High	Quasi-real-time monitoring, depending on cell live time	DNA staining and advanced image analysis	33, 34
Luciferase (LUX) Assay	Loss of signal indicates growth inhibition by antibiotics	No	**Yes**	Fluorescence	**Low**	Quasi-real-time monitoring, depending on cell live time	Genetic engineering of strains	35 – 37
ATP Bioluminescence Assay	Measures ATP levels as proxy for viable cells after antibiotic exposure	No	**Yes**	Fluorescence	**Low**	2-6 hours	Specialized reagents	36 – 39
Flow Cytometry	Fluorescent dyes distinguish live/dead cells after antibiotic treatment	No	**Yes**	Fluorescence	High	2-6 hours	Specialized equipment and labelling	36, 40, 41
Automated Broth Microdilution	Automated serial dilutions in microtiter plates, growth monitored by optical density	No	No	Impacted area of bacteria on plate	High	6-24 hours after initial isolation	Expensive equipment	36, 42 – 44
Broth Dilution	Bacteria exposed to serial dilutions of antibiotic in broth, MIC is lowest concentration inhibiting growth	No	No	Impacted area of bacteria on plate	**Low**	18-24 hours, (or 48 hours, including prior bacterial isolation and identification)	**No**	36, 37, 44 – 46
Agar Diffusion (Kirby-Bauer, E-test)	Antibiotic diffusion from a disk/strip creates an inhibition zone on agar plate with bacterial lawn, standard method	No	No	Impacted area of bacteria on plate	**Low**	18-24 hours, (or 48 hours, including prior bacterial isolation and identification)	**No**	36, 37, 43, 44, 47
**Microbubble Printing (this work)**	Massive microbubbles capture and print nanosensor/bacterial hybrids into arrays	**Yes**	**Yes**	**Raman of released metabolites**	**Low, similar to fluorescent microscope**	**Quasi-real time monitoring, depending on cell live time (20 min to 2-3 hrs)**	**No**	This work

21. Supplementary Table S5. Raman peak positions of purine derivatives for identification [48]

Purine derivative	Literature Value
Hypoxanthine	550, 624, 692, 725, 920, 963, 1027, 1088, 1293, 1330, 1365, 1396, 1457, 1527, 1586, 1726
Xanthine	512, 573, 659, 881, 960, 1053, 1132, 1244, 1317, 1368, 1479, 1545, 1577, 1700
Guanine	489, 527, 578, 669, 880, 963, 1058, 1139, 1168, 1205, 1261, 1294, 1351, 1382, 1467, 1539, 1577, 1641
Uric Acid	498, 594, 545, 743, 813, 897, 1081, 1141, 1400, 1504, 1572, 1639
AMP	550, 620, 645, 673, 737, 903, 967, 1033, 1074, 1131, 1238, 1322, 1395, 1470, 1581, 1647
Adenine	559, 629, 681, 737, 785, 912, 968, 1033, 1188, 1234, 1274, 1320, 1344, 1379, 1400, 1455, 1548

22. Supplementary Note 1: Heating and simulation and laser heating

While many works utilize heat-induced thermophoretic and accompanying Marangoni flows to attract and trap particles onto the surface,[8,49,50] the heat generation in our method in contrast, is minimal. Due to the low intensity, heating due to laser absorption is very small at the surface of the Si and the ohmic heating in our experiments is similarly minimal. Simulations (**Figure S2**) and calculations indicate a heat increase of less than <<1 K for each source, due to the high photoconductivity of our substrate and the low laser intensity requirement to generate a bubble. Here, we provide the parameters that were used in the ohmic heating and laser heating simulations:

Description	Expression	Reference
**Thermal conductivity of a-Si**	$1.3 \times 10^{-11}(T-900)^3 + 1.3 \times 10^{-9}(T-900)^2 + 10^{-6}(T-900) + 10^{-2}$ [W/cm·K]	51,52
**Specific heat of a-Si**	0.171 T / 1865 K + 0.952 [J/g·K]	
**Density of a-Si**	2260 [kg/m^3]	
**Reflectivity of solid a-Si**	0.58	
**Absorption coefficient of a-Si**	$5.02 \times 10^3 \exp(T/430)$ [/cm]	

The temperature increase due to the absorption of light in the a-Si layer was simulated using COMSOL Multiphysics and further details are provided in the main text of the manuscript.

23. Supplementary Note 2: Effect of H_2O_2 and CTAC

We explore how various additives and surfactants such as H_2O_2 and cetyltrimethylammonium chloride (CTAC) and a thin Pt catalyst layer on the counter electrode may affect the minimum laser intensity. Under normal conditions (a 0.5 M Na_2SO_4 solution with a -5 V DC bias superimposed on a 12 V_{pp} AC E-field with a frequency of 1.5 kHz), the average minimum laser intensity is 390.1 ± 17.5 mW/cm^2. With the addition of H_2O_2, this number falls to 288.4 ± 36.1 mW/cm^2, which represents a 26% drop in the required laser intensity. Despite having a slightly higher surface tension than water with a value of 80.4 mN/m, which would prohibit bubble formation, H_2O_2 decreases the required laser intensity due to two factors: 1) H_2O_2 has an acid dissociation constant (pKa) of 11.6, meaning that it is slightly acidic in nature; releasing H^+ ions into the solution which is the basis for the hydrogen bubble generation by the E-field. 2) H_2O_2 has been reported as an effective hole scavenger and can prevent hole-electron recombination during illumination.[53,54] Indeed, as can be seen in equation (1), the increase of recombination lifetime leads to an increase of the photocurrent of amorphous silicon, leading to smaller minimum intensity values.

CTAC, on the other hand, acts as a surfactant that reduces the surface tension of water at the liquid-gas interface, which can facilitate bubble growth. It has been reported that the addition CTAC can reduce the surface tension to 36.1 mN/m, compared to the surface tension of plain water of 71.78 mN/m.[55] For our experiments, we use a concentration of 1250 mg/L to ensure the highest decrease of surface tension. Indeed, as can be seen in **Figure 2f**, the addition of CTAC has shown to greatly decrease the required laser intensity down to 219.7 ± 51.1 mW/cm^2. The surfactant reduces surface tension and facilitates the ease with which microbubbles are formed. We expect that other surfactants, such as anionic or non-ionic

surfactants, would similarly facilitate the reduction of the MRLI. PBS was evaluated to demonstrate that the electrochemical microbubble method is compatible with solutions commonly used in cell cultures. We did not directly compare PBS with solutions containing hydrogen peroxide or CTAC due to differences in ionic species and concentration, which would lead to different electrical impedances, making a direct comparison of electrochemical bubble behavior less meaningful. Notably, PBS has a lower overall ionic concentration than the other control solutions. To compensate for this, we applied a higher DC bias, a lower frequency, and used a thinner PDMS well. These adjustments effectively reduced the required minimum laser intensity, at times even below that needed for higher ionic strength solutions containing hydrogen peroxide or CTAC.

24. Supplementary Note 3: Considerations on the formation of single vs multiple bubbles

A single larger bubble possesses a smaller surface area compared to multiple smaller bubbles that contain the same overall volume of gas, and hence minimization of the overall surface energy favors the formation of a single bubble. Hydrophobic or partially hydrophobic nanoparticles are known to attach themselves to gas bubbles and can in turn stabilize the bubbles. Smaller bubbles possess a larger curvature and are most probably not as well covered by nanoparticles, such that these smaller bubbles fuse to form larger, more stable 'single' bubbles. The exact mechanism of formation and hence the resulting size is likely to be a complex process that is an interplay between the stabilizing effect of the nanoparticles, surface tension effects, as well as pressure-volume work and entropy considerations. A full exploration of all of the relevant parameters is beyond the scope of this work.

25. Supplementary Note 4: Shrinkage of the H_2 bubbles

The shrinkage and collapse of the bubbles are the result of competition between the outward H_2 diffusion pressure and the inward pressure of the surrounding liquid at the gas-liquid interface. Most of the bubbles show a similar trend, with an initial slow shrinking speed likely due to dynamic interactions with supersaturated hydrogen at the bubble surface vicinity, which is followed by a rapid collapse (**Figure S15a**). Here, most of the bubbles eventually shrank and collapsed under ~80 s. However, some of the bubbles exhibited a secondary expansion phase even after the E-field and laser pattern is turned off (**Figure S15b**). This additional growth of the bubbles increases the bubble lifetime, taking the total time to shrink and collapse from ~ 125 s to over 300 s. Similar bubble growth has been found in bubbles in supersaturated water at the vicinity of an electrode.[56]

26. Supplementary Note 5: Consideration of the surface state and particle density

The versatile bubble-patterning technique is applied for capturing and assembling colloidal nanoparticles. For this purpose, it is crucial to control the surface state of the substate, as well as the shape and size of microbubbles.

1) On a native α-Si:H surface, a hydrogen bubble maximizes its contact and adapts to a truncated sphere due to the hydrophobic nature of both the hydrogen gas and the α-Si:H.[11,57] As a result, the particles deposit into a coffee ring structure (**Figure S4**). We, therefore, tune the α-Si:H substrate to be hydrophilic to obtain single-spot particle deposition via a spherical, single-contact microbubbles.

2) When the particle density inside the solution is low, or no particles are inside the solution, multiple, instead of single, bubbles form under the light spots (**Figure 4a, inset**, **Video S5**),[11] which is not suitable for precisely positioning nanoparticles. The single-bubble formation in a nanoparticle colloid suspension (**Video S5**), in contrast to multi-bubbles made without nanocolloids, could be attributed to a total energy reduction driven by surface-area minimization as discussed in Note 3.

27. Supplementary Note 6: Gas-diffusion controlled process of microbubble growth

While a slope of 0.5 is often assumed for ideal diffusion-controlled growth,[58,59] variations from the diffusion-controlled growth model may exist due to the following factors: 1) The research in references[58,59] used solutions such as sulfuric acid in water, which exhibits a higher hydrogen ion concentration for hydrogen bubble growth compared to that in D.I. water used in our study. 3) The model assumes that the bubble is far away from any solid wall since the surface roughness may alter the bubble growth.[60] While, we grew bubbles next to a substrate. 4) Last, we usually grew bubbles in a closely spaced array. It has been reported that the presence of close-by bubbles decreases a bubble's growth.[61] The reported power factor of a second bubble generated next to an already existing bubble is 0.44, which exactly agrees with that we observed in experiments.

28. Supplementary Note 7: Measurement of *B. Subtilis* metabolites using SERS

For control, we measure 1) SERS of particle aggregates without any bacterial cells (**Figure S11c**), 2) SEM and SERS of PS-Ag / cells formed in islands in an antibiotic solution (**Figure S16**), and 3) SERS of bubble-deposited PS-Ag /cells in DI water (**Figure S10**). The peaks measured in 1) were noted and not considered for metabolite identification in SERS of *B. Subtilis*. Measurements from 2) show that even after two hours in DI water the SERS peaks are still present, compared to those exposed to antibiotics where the peaks disappear after a certain amount of time. When a larger number of particle/cells are deposited, as can be seen in 3), a larger number of peaks is observed. As noted in the main manuscript, we observed that the metabolite signals last longer for a higher population, which may be the result or the combination of the following: with higher cell densities, 1) the physical cell surface area exposed to the antibiotic solution is reduced, 2) there is a higher possibility of the existence of drug-resistant cells, and 3) cells exhibit collective antibiotic resistance via cell-cell communication, increasing survival when exposed to antibiotics.[62] The peaks for purine derivatives such as hypoxanthine, xanthine, guanine, AMP, uric acid and adenine are identified and used in this study (**Table S5**).[48]

To confirm that the average SERS degradation time distributions are significantly different for each condition, we use the Mann-Whitney U test by using data (the time for the last metabolite peak to

disappear from each measurement) from **Figure 7**, **Figure S14**, and **Figure S16**. The calculated z values for each comparison, the bacterial island-large clusters, large/small clusters, and bacterial island-small clusters, are -1.98, -2.78, -2.72, respectively. As these values greatly fall outside of the critical values ± 1.96 at a significance level of 0.05, we conclude that the differences in SERS degradation times are statistically significant.

Materials

200 nm fluorescent PS spheres used for particle deposition were purchased from Thermofisher Scientific. The carboxylate-terminated spheres have a surface charge of 0.3699 meq / g (technical data sheet). Sodium sulfate (anhydrous) and hydrogen peroxide (30%) were purchased from Fisher Chemical. Non-functionalized silver nanoparticles (citrate capped, 40 nm) were purchased from NNCrystal US Corporation and washed with DI-water via centrifugation 3x and concentrated 4x before use. *E-coli* (K12 strain) was purchased from Carolina Biological Supply Company. *Bacillus subtilis* (6051) and CHO cells (CCL-61) were purchased from ATCC. Polydimethylsiloxane (Sylgard 184 silicone elastomer kit) was purchased from Dow Corning Corporation. Cetyltrimethylammonium chloride (25 wt. % in H_2O), Poly(diallyldimethylammonium chloride) (average Mw 200,000-350,000, 20 wt. % in H2O), RPMI-1640 Medium (R8758), Fetal Bovine Serum (F2442), Penicillin-Streptomycin (P4333), and Trypsin-EDTA solution (T4049) were purchased from Sigma Aldrich. All reagents were used without further purification. FTO (Fluorine-doped Tin Oxide) glass (0.7 mm thickness and 13 – 15 Ohm/Sq sheet resistance), purchased from MSE Supplies (Product TEC 15), was used as the counter electrode. Sapphire glass–FTO glass–Hydrogen-terminated silicon substrates were fabricated at the University of Stuttgart.

Equipment

The experiments are carried out on an inverted microscope (Olympus IX 70) equipped with a 532 nm laser (DJ532-40, Thorlabs) and a digital light projecting device (DLP, Model DLi4130 .7"VIS XGA, Digital Light Innovations). Scanning electron microscopy (Model Quanta 650, FEI, Model Apreo 2 SEM, Thermo Fisher Scientific) was used for the characterization of deposited particles. A function generator (Model 33250A, Agilent), coupled with a custom-made amplification circuit was used to create AC/DC fields with the desired electric field intensity. A reactive ion etcher (Model RIE-1C, Samco) or UVO-cleaner (Model 30, Jelight) was used to clean the surface of the silicon sample and etch the deposited PS spheres. The etcher was also used to etch away part of the silicon so that the FTO glass underneath could be exposed for electric connections. An e-beam and sputtering system (Model PVD75, Kurt J. Lesker Company) was used to deposit a thin film of Pt on top of a FTO-glass counter electrode substrate to test the effects on bubble generation. A 532 nm wavelength laser (Model Sapphire, Coherent) was used for the characterization of minimum laser intensity, and another 532 nm wavelength laser (DJ532-40, Thorlabs), coupled with a digital light projector (Model DLi4130 .7"VIS XGA, Digital Light Innovations) was used for light pattern projection.

References:

1. Bertin, N. *et al.* Bubble-based acoustic micropropulsors: active surfaces and mixers. *Lab Chip* **17**, 1515–1528 (2017).

2. Conde, A. J., Keraite, I., Ongaro, A. E. & Kersaudy-Kerhoas, M. Versatile hybrid acoustic micromixer with demonstration of circulating cell-free DNA extraction from sub-ml plasma samples. *Lab Chip* **20**, 741–748 (2020).

3. Ahmed, D. *et al.* Acoustofluidic chemical waveform generator and switch. *Anal. Chem.* **86**, 11803–11810 (2014).

4. Jeong, J., Jang, D., Kim, D., Lee, D. & Chung, S. K. Acoustic bubble-based drug manipulation: Carrying, releasing and penetrating for targeted drug delivery using an electromagnetically actuated microrobot. *Sens. Actuators A Phys.* **306**, 111973 (2020).

5. Xie, Y. *et al.* Probing Cell Deformability via Acoustically Actuated Bubbles. *Small* **12**, 902–910 (2016).

6. Hu, W., Fan, Q. & Ohta, A. T. An opto-thermocapillary cell micromanipulator. *Lab Chip* **13**, 2285–2291 (2013).

7. Zhao, C. *et al.* Theory and experiment on particle trapping and manipulation via optothermally generated bubbles. *Lab Chip* **14**, 384–391 (2014).

8. Lin, L. *et al.* Bubble-Pen Lithography. *Nano Lett.* **16**, 701–708 (2016).

9. Meng, L. *et al.* Microbubble enhanced acoustic tweezers for size-independent cell sorting. *Appl. Phys. Lett.* **116**, 073701 (2020).

10. Ma, Z. *et al.* Spatial ultrasound modulation by digitally controlling microbubble arrays. *Nat. Commun.* **11**, 4537 (2020).

11. Ma, Z., Joh, H., Fan, D. E. & Fischer, P. Dynamic Ultrasound Projector Controlled by Light. *Adv. Sci.* **9**, 2104401 (2022).

12. Moo, J. G. S., Presolski, S. & Pumera, M. Photochromic Spatiotemporal Control of Bubble-Propelled Micromotors by a Spiropyran Molecular Switch. *ACS Nano* **10**, 3543–3552 (2016).

13. Li, Y. *et al.* Light-controlled bubble propulsion of amorphous TiO2/Au Janus micromotors. *RSC Adv.* **6**, 10697–10703 (2016).

14. Rao, D. C. K., Mooss, V. S., Mishra, Y. N. & Hanstorp, D. Controlling bubble generation by femtosecond laser-induced filamentation. *Sci. Rep.* **12**, 15742 (2022).

15. Lin, L. *et al.* Bubble-Pen Lithography. *Nano Lett.* **16**, 701–708 (2016).

16. Xiao, P., Li, W. J. & Du, R. Micro-bubble generation with micro-watt power using carbon nanotubes heating elements. *2007 7th IEEE Conference on Nanotechnology (IEEE NANO)* 983–988 (IEEE, 2007).

17. YASUDA, T., TAKAHASHI, N., BABA, M., TEI, K. & YAMAGUCHI, S. An Experimental Study on Micro-Bubble Generation by Laser-Induced Breakdown in Water. *Rev. Laser Eng.* **36**, 1273–1275 (2008).

18. Xiao, P. & Staack, D. Microbubble generation by microplasma in water. *J. Phys. D: Appl. Phys.* **47**, 355203 (2014).

19. Afrizal, A. *et al.* Anaerobic single-cell dispensing facilitates the cultivation of human gut bacteria. *Environ. Microbiol.* **24**, 3861–3881 (2022).

20. Yin, J. *et al.* A droplet-based microfluidic approach to isolating functional bacteria from gut microbiota. *Front. Cell. Infect. Microbiol.* **12**, 920986 (2022).

21. Inoue, I., Wakamoto, Y., Moriguchi, H., Okano, K. & Yasuda, K. On-chip culture system for observation of isolated individual cells. *Lab Chip* **1**, 50-55 (2001).

22. Marcy, Y. *et al.* Nanoliter Reactors Improve Multiple Displacement Amplification of Genomes from Single Cells. *PLoS Genet.* **3**, e155 (2007).

23. Marie, R. *et al.* Sequencing of human genomes extracted from single cancer cells isolated in a valveless microfluidic device. *Lab Chip* **18**, 1891–1902 (2018).

24. Van Strijp, D. *et al.* Complete sequence-based pathway analysis by differential on-chip DNA and RNA extraction from a single cell. *Sci. Rep.* **7**, 11030 (2017).

25. Dey, S. S., Kester, L., Spanjaard, B., Bienko, M. & van Oudenaarden, A. Integrated genome and transcriptome sequencing of the same cell. *Nat. Biotechnol.* **33**, 285–289 (2015).

26. Hou, Y. *et al.* Single-cell triple omics sequencing reveals genetic, epigenetic, and transcriptomic heterogeneity in hepatocellular carcinomas. *Cell Res.* **26**, 304–319 (2016).

27. Gerlach, Jan. P. *et al.* Combined quantification of intracellular (phospho-)proteins and transcriptomics from fixed single cells. *Sci. Rep.* **9**, 1469 (2019).

28. Grützkau, A. & Radbruch, A. Small but mighty: How the MACS ® -technology based on nanosized superparamagnetic particles has helped to analyze the immune system within the last 20 years. *Cytom. Part A* **77**, 643–647 (2010).

29. Li, M. & Anand, R. K. High-Throughput Selective Capture of Single Circulating Tumor Cells by Dielectrophoresis at a Wireless Electrode Array. *J. Am. Chem. Soc.* **139**, 8950–8959 (2017).

30. Park, J. Y. *et al.* Single cell trapping in larger microwells capable of supporting cell spreading and proliferation. *Microfluid. Nanofluidics* **8**, 263–268 (2010).

31. Collins, D. J. *et al.* Two-dimensional single-cell patterning with one cell per well driven by surface acoustic waves. *Nat. Commun.* **6**, 8686 (2015).

32. Guo, F. *et al.* Three-dimensional manipulation of single cells using surface acoustic waves. *Proc. Natl. Acad. Sci. USA* **113**, 1522–1527 (2016).

33. Siqueira-Neto, J. L. *et al.* An image-based high-content screening assay for compounds targeting intracellular leishmania donovani amastigotes in human macrophages. *PLoS Negl. Trop. Dis.* **6**, e1671 (2012).

34. Peach, K. C., Bray, W. M., Winslow, D., Linington, P. F. & Linington, R. G. Mechanism of action-based classification of antibiotics using high-content bacterial image analysis. *Mol. Biosyst.* **9**, 1837–1848 (2013).

35. Billard, P. & Dubow, M. S. Bioluminescence-Based Assays for Detection and Characterization of Bacteria and Chemicals in Clinical Laboratories. *Clin. Microbiol. Rev.* **31**, 1-14 (1998).

36. Lage, O. M. *et al.* Current screening methodologies in drug discovery for selected human diseases. *Marine Drugs* **16**, 279 (2018).

37. Bento, C. M., Gomes, M. S. & Silva, T. Evolution of antibacterial drug screening methods: Current prospects for mycobacteria. *Microorganisms* **9**, 2562 (2021).

38. Kapoor, R. & Yadav, J. S. Development of a rapid ATP bioluminescence assay for biocidal susceptibility testing of rapidly growing mycobacteria. *J. Clin. Microbiol.* **48**, 3725–3728 (2010).

39. Cai, Y. *et al.* Using an adenosine triphosphate bioluminescent assay to determine effective antibiotic combinations against carbapenem-resistant gram negative bacteria within 24 hours. *PLoS One* **10**, e0140446 (2015).

40. Wong, F. H.-S. *et al.* Determining the development of persisters in extensively drug-resistant Acinetobacter baumannii upon exposure to polymyxin B-based antibiotic combinations using flow cytometry. *Antimicrob. Agents Chemother.* **64**, e01712-19 (2020).

41. Saint-Ruf, C. *et al.* Antibiotic susceptibility testing of the gram-negative bacteria based on flow cytometry. *Front. Microbiol.* **7**, 1121 (2016).

42. Smith, K. P. & Kirby, J. E. Verification of an automated, digital dispensing platform for at-will broth microdilution-based antimicrobial susceptibility testing. *J. Clin. Microbiol.* **54**, 2288–2293 (2016).

43. Hoelzer, K. *et al.* Agar disk diffusion and automated microbroth dilution produce similar antimicrobial susceptibility testing results for salmonella serotypes Newport, Typhimurium, and 4,5,12:i-, but differ in economic cost. *Foodborne Pathog. Dis.* **8**, 1281–1288 (2011).

44. Gajic, I. *et al.* Antimicrobial Susceptibility Testing: A Comprehensive Review of Currently Used Methods. *Antibiotics* **11**, 427 (2022).

45. Swenson, J. M., Killgore, G. E. & Tenover, F. C. Antimicrobial susceptibility testing of Acinetobacter spp. by NCCLS broth microdilution and disk diffusion methods. *J. Clin. Microbiol.* **42**, 5102–5108 (2004).

46. Wiegand, I., Hilpert, K. & Hancock, R. E. W. Agar and broth dilution methods to determine the minimal inhibitory concentration (MIC) of antimicrobial substances. *Nat. Protoc.* **3**, 163–175 (2008).

47. Blanscet, M. L., Tordik, P. A. & Goodell, G. G. An Agar Diffusion Comparison of the Antimicrobial Effect of Calcium Hydroxide at Five Different Concentrations with Three Different Vehicles. *J. Endod.* **34**, 1246–1248 (2008).

48. Premasiri, W. R. *et al.* The biochemical origins of the surface-enhanced Raman spectra of bacteria: a metabolomics profiling by SERS. *Anal. Bioanal. Chem*. **408**, 4631–4647 (2016).

49. Gargiulo, J., Cerrota, S., Cortés, E., Violi, I. L. & Stefani, F. D. Connecting Metallic Nanoparticles by Optical Printing. *Nano Lett.* **16**, 1224–1229 (2016).

50. Armon, N. *et al.* Continuous Nanoparticle Assembly by a Modulated Photo-Induced Microbubble for Fabrication of Micrometric Conductive Patterns. *ACS Appl. Mater. Interfaces* **9**, 44214–44221 (2017).

51. Jellison, G. E. & Modine, F. A. Optical absorption of silicon between 1.6 and 4.7 eV at elevated temperatures. *Appl. Phys. Lett.* **41**, 180–182 (1982).

52. Förster, J. & Vogt, H. Excimer Laser-Annealing of Amorphous Silicon Layers. *Simulation* **88**, 90 (2011).

53. Dotan, H., Sivula, K., Gr, M. & Warren, S. C. Probing the photoelectrochemical properties of hematite (a-Fe2O3) electrodes using hydrogen peroxide as a hole scavenger. *Energy Environ. Sci.* **4**, 958–964 (2011).

54. Sreedhar, A. *et al.* Highly supportive hydrogen peroxide as a hole scavenger to improve the visible light water splitting activity of flake-like Co-doped ZnO thin films. *Sol. Energy* **191**, 151–160 (2019).

55. Chen, G. *et al.* Preparation and surface activity study of amino acid surfactants. *C. R. Chim.* **22**, 277–282 (2019).

56. Chandran, P., Bakshi, S. & Chatterjee, D. Study on the characteristics of hydrogen bubble formation and its transport during electrolysis of water. *Chem. Eng. Sci.* **138**, 99–109 (2015).

57. Sakuma, G., Fukunaka, Y. & Matsushima, H. Nucleation and growth of electrolytic gas bubbles under microgravity. *Int. J. Hydrogen Energy* **39**, 7638–7645 (2014).

58. Westerheide, D. E. & Westwater, J. W. Isothermal growth of hydrogen bubbles during electrolysis. *AIChE J.* **7**, 357–362 (1961).

59. Higuera, F. J. A model of the growth of hydrogen bubbles in the electrolysis of water. *J. Fluid Mech.* **927**, A33 (2021).

60. Aoki, K. J. Frequency-dependence of electric double layer capacitance without Faradaic reactions. *J. Electroanal. Chem.* **779**, 117–125 (2016).

61. Choi, H., Li, C. & Peterson, G. P. Dynamic Processes of Nanobubbles: Growth, Collapse, and Coalescence. *J. Heat Transfer* **143**, 102501 (2021).

62. Vega, N. M. & Gore, J. Collective antibiotic resistance: Mechanisms and implications. *Curr. Opin. Microbiol.* **21**, 28-34 (2014).
