# Supplementary material for: Massively parallel microbubble nano-assembly
Source: Nat Commun. 2025 Jul 22;16:6743. doi: 10.1038/s41467-025-62070-9 (PMC12284250; doi:10.1038/s41467-025-62070-9)
Supplement: Supplementary file 2 — Description of Additional Supplementary Files [file 41467_2025_62070_MOESM2_ESM.pdf]

All videos are in real time except when indicated otherwise.

File name: Supplementary Video 1

Description: Summary: precise and complex bubble formation, a running horse animation, bubble-based particle deposition, and bacterial sensing application.

File name: Supplementary Video 2

Description: A Longhorn logo overlayed on the map of Texas; and Minerva. Microbubbles are simultaneously generated in 'one-shot' by the corresponding light pattern. The last video frame is displayed for an additional two seconds to aid the viewer.

File name: Supplementary Video 3

Description: A running-horse animation displayed by light-directed microbubbles.

File name: Supplementary Video 4

Description: A continuously expanding circle made of light-triggered microbubbles and a display of a firework by the corresponding light-driven microbubble pattern (repeated 2x).

File name: Supplementary Video 5

Description: Comparison of bubble formation with and without nanoparticles in solution; laser spot size: 10 μm and 18 μm.

File name: Supplementary Video 6

Description: Deposition of particles in an array on a PDDA functionalized surface. The last video frame is displayed for an additional two seconds to aid the viewer.

File name: Supplementary Video 7

Description: Deposition of particles using self-assembled microbubbles generated by a simple circular pattern. The last video frame is displayed for an additional two seconds to aid the viewer.
