# Supplementary material for: Massively parallel microbubble nano-assembly
Source: Nat Commun. 2025 Jul 22;16:6743. doi: 10.1038/s41467-025-62070-9 (PMC12284250; doi:10.1038/s41467-025-62070-9)
Supplement: Supplementary file 10 — Transparent Peer Review file [file 41467_2025_62070_MOESM10_ESM.pdf]

nature portfolio

Peer Review File

Massively Parallel Microbubble Nano-Assembly

Corresponding Author: Professor Donglei Fan

This file contains all reviewer reports in order by version, followed by all author rebuttals in order by version.

Version 0:

Reviewer comments:

Reviewer #1

(Remarks to the Author)
General Comments: The authors describe a clever system to produce bubbles at scale in well-defined patterns using an opto-electrochemical method. The authors seem to have a good fundamental understanding of the working mechanism behind bubble formation, and they provide useful optimization results to highlight effects from reagents to lower redox voltage and surface tension toward the goal of forming bubbles with lower light intensity or with greater stability. Finally, the authors demonstrate several use cases of the bubbles to drive clustering of various colloidal particles in solution toward an example of how this system could be used in applications. Overall, the results are well-presented and described. I support publication of this work in NC, barring a few minor clarifications.

Specific Comments:
• The authors should elaborate on their selection of CTAC surfactant as opposed to anionic or non-ionic surfactants.
• Why does PBS perform better than hydrogen peroxide- or CTAC-only controls?
• Figure 2: The authors should specify in the caption or figure what the AC frequency was in 2C to which a DC bias was applied.
• Terminology: Do the examples of nanocrystal, EV, polymer nanosphere, and bacteria concentration really constitute assembly? Assembly in the colloidal sense usually implies some level of order amongst is being created its constituent components. I would call the structures formed aggregates (as the authors do on a few occasions), agglomerates, or clusters. Please define what is meant by assembly in this bubble-mediated context.
• While the bubbles rest against the ceiling of the fluid chamber, it is not clear what happens after particle assembly. Do the complexes gain enough density to settle? If so, how does this affect the morphology of the clusters? Please discuss.
• Figure 7: The explanation of data in 7C-D is insufficient both in the results section and in the figure caption. In 7C (large clusters) and 7D (small clusters), there are 4 graphs with multiple measurements over time. What does each graph correspond to? Please discuss and add some level of interpretation of these results in the results section.
• Additionally, were control experiments performed in Figure 7 (i) without cell assembly and (ii) without vancomycin? These types of controls are crucial for understanding the benefit of this bubble-concentration approach in biological assays. This is my biggest concern with this paper.
• Did the authors apply any statistical test to Figure 7e (e.g., paired T test)?

Reviewer #2

(Remarks to the Author)
Summary:
The authors present an innovative opto-electrochemical method to generate highly defined microbubble patterns, enabling the assembly of nanocrystals, extracellular vesicles, polymer nanospheres, and live bacteria into complex patterns with high precision (down to 2 µm spatial resolution). Their method integrates light-based projection with low-energy electrolysis, based on a photoconducting α-Si:H surface, allowing the controlled formation of microbubbles via electrolysis upon localized illumination. By modifying the α-Si:H surface and introducing nanoparticles, they successfully suppress undesired clustering and enable individual light-addressable bubbles across a broad size range.

This work introduces a novel platform that enables dynamic, scalable, low-temperature, and high-resolution microbubble patterning — addressing major limitations of both photothermal and template-based methods. The integration of optical and electrochemical control mechanisms is a noteworthy contribution with potential for wide-ranging applications.

Originality and significance:
• The method appears innovative because it allows simultaneous generation of a large number of microbubbles and direct formation of complex shapes at room temperature, making it suitable for temperature-sensitive biological samples.
• Other methods have the limitations that the available bubble patterns are static (when prefabricated templates are used), or that the direct formation of a microbubble with a laser requires high intensities and temperatures.

This work is likely to attract considerable interest within the micromanipulation community, as the presented method addresses several longstanding limitation regarding throughput, spatial precision, and biocompatibility. The ability to dynamically project arbitrary patterns in one shot even at low power and ambient temperatures offers significant advantages in terms of scalability, versatility, and suitability for applications in biosensing and cell-based assays.
Methodology:
The experiments are well designed, executed and analyzed and strongly support the drawn conclusions. In particular, the hypothesis of an electrochemical (rather than thermal) mechanism of bubble formation is well supported.
However, the manuscript would benefit from improved clarity in certain sections, more rigorous justification of the biological proof-of-concept, and greater attention to data visualization. Specific recommendations are outlined below.

Detailed comments:
Clarity and context:
1. The research goal and the specific challenge being addressed were not immediately clear. A more explicit statement of the intended application early on (e.g., creation of cell-sensor assays with single- or few-cell resolution) would improve clarity. Overall strengthening of the “problem-current technological limitations-solution narrative” would enhance understanding of the potential impact and implications to a broader audience, such as that of Nat Comm
2. Especially the abstract, but also the introduction could benefit from more clarity. The authors should emphasize the novelty of integrating optical and electrochemical techniques in patterning applications. Optically induced bubble patterning has been done before (studies have demonstrated the use of laser-induced microbubbles for precise material deposition ACS Applied Materials & Interfaces (this work is also cited)), and electrochemical bubble formation has also been done before, but the integration of optical and electrochemical techniques for bubble formation, which enables one shot assembly is novel
3. Justification of Biological Demonstrations and which general implications can be draw from it: The application of the platform to metabolic profiling and antibiotic response in bacteria is intriguing but insufficiently motivated. The rationale for choosing these particular biological systems is not provided. How do they showcase the unique capabilities of the platform?
4. The discussion would benefit from more critical reflections on their own work? For example, how stable are the patterns? How sensitive to perturbations?
5. Specific lines needing clarification:
o Line 20: The term “parallel” is ambiguous. Clarify whether this refers to the simultaneous formation of multiple microbubbles as opposed to single bubble-based techniques.
o Line 44: The transition to applications in cell-sensor assays is abrupt. Provide a smoother contextual link to explain the relevance of microbubble patterning in this domain.
o Lines 49–51, 219–220: The choice of bacterial profiling as a demonstration lacks explanation. A brief rationale would improve the impact of this section.
o Line 158–160: Clarify whether the authors recommend using only PBS for biological applications to mitigate the potential toxicity of H_2O_2 and CTAC.
o Line 173, Figure 4, Line 276: Specify the type, size, and concentration of nanoparticles used. Are these parameters critical to the success of the approach?
o Line 299: spelling error. Chinese Hamster Ovary

Figures & Data Representation:
1. General comment: The current color schemes are visually overwhelming, with an excessive number of distinct colors used within single plots. A more restrained and visually coherent palette would improve both the aesthetic quality and interpretability of the figures.
2. Figure 4b: missing legend for the different colors and symbols of the many (10?) line plots that are displayed to show the relation between radius and time
3. Figures 7c, d: Excessive color diversity without concrete meaning. Consider simplifying the palette, i.e. shades of a color to indicate time progression over 120 min or 180 min
4. Figures 7c, d & S14-S16: The use of "m" for minutes is unconventional and could be confused with meters as SI unit. Replace “m” with “min” for clarity.
5. Figure S10: The insert with labels for time points (0 min, 30 min, etc.) should be larger for readability. Also, replace “m” with “min” for clarity.
6. Figure S11c & S15: The meaning of different colors is unclear and should be explained.

Reviewer #3

(Remarks to the Author)
I co-reviewed this manuscript with one of the reviewers who provided the listed reports. This is part of the Nature Communications initiative to facilitate training in peer review and to provide appropriate recognition for Early Career Researchers who co-review manuscripts.

Reviewer #4

(Remarks to the Author)
The authors report an opto-electrochemical method to rapidly and controllably generate arrays of microbubbles for various purposes. Produced bubbles range in size from a few to over one hundred micrometers in size with positioning accuracy of ~2 micrometers. Biocompatibility is proven by demonstrating that the low required light intensity does not harm cells, bacteria, and sensitive nanomaterials. After fully characterizing the bubble generation process, the authors demonstrate how nanosensor-bacterial cell arrays can be used for spectroscopic profiling of metabolites to evaluate the antibiotic response of bacterial assemblies.

The authors effectively establish the need for controllable microbubble generation, primarily for acoustofluidic applications. Existing methods are presented to highlight specific shortcomings (e.g., the static nature of templates, and the thermal damage induced by conventional opto-thermal approaches). These shortcomings motivate the introduction of electrolysis for bubble production, and a creative electrode configuration (including a photoconducting alpha-Si:H layer that facilitates light-based bubble formation and growth) enables dynamic, controllable, and biocompatible microbubble arrays. The primary application of the approach appears to be for formation of particle aggregates, either nanomaterials, cells, or both and then analysis of those aggregates (as opposed to the aforementioned acoustofluidic applications). To this end, the authors provide various demonstrations, including the profiling of bacterial metabolites after exposure to antibiotics. The work is complete, the manuscript is clearly written, and the demonstrations are compelling. For these reasons, I believe the manuscript is suitable for publication in Nature Communications after addressing the mostly minor points below:

1. Introduction: as mentioned above, the alternate methods for microbubble production are primarily introduced with reference to their applications in acoustofluidics (e.g., for shaping ultrasound fields, pumping, mixing, etc.). The reported method is primarily used for nanomaterial deposition and cell aggregation. Is there an inherent difference between previous methods and the present method that makes these methods more or less suited for particular applications, or were the demonstrations chosen based on perceived greater impact? Put another way, would the current method work just as well as other methods for acoustofluidic applications?
2. Working mechanism…: the fabrication process of the enabling electrode configuration is well-described, and the method of optical activation of the local electric field is very creative. Has alpha-Si:H been used for a similar purpose (not to produce microbubbles, but just to activate the field locally)? This is purely from curiosity.
3. Working mechanism…: is there an error in the text line 101? Should 7.4 W/cm2 be 0.74 W/cm2? It appears from Fig. 2b that the intensity decreases below 1000 mW/cm2 at the lowest frequency (which would be 740 mW/cm2). On that note, is there a reason for not using W/cm2 as the y-axis in Fig. 2b?
4. Working mechanism…: the plots in Fig. 2 have a couple of issues, i) the fonts are generally too small, particularly for the insets, which are not possible to read, ii) it might be better to have the inset for 2a as its own panel, since it is a different variable, and iii) the inset for 2b needs to make clear that the y-axis is the same as for the main plot. Generally, axes should be labeled.
5. Working mechanism…: in line 103, it says “MRLI is further reduced ~2.72-fold with an increasing superimposed DC bias from -3.5 to -5.5 V”. This is a bit misleading as it is not a “further” increase over the frequency dependence result. It is a separate result given that the frequency result was found at a bias of -5.0 V. Perhaps this is just word usage, but it would be clearer to say, “Further, the MRLI is reduced…” or “The MRLI is also reduced by increasing the superimposed DC bias…”.
6. Working mechanism…: in lines 143-144, the authors argue that the AC E-field is studied due to “its importance in understanding the working mechanism…” before mentioning robust bubble creation. The authors then spend a lot of time discussing surface degradation in DC vs. AC fields. Why is the AC E-field important to understand the working mechanism? Versus purely just avoiding degradation?
7. Working mechanism…: related to “6”, in line 152, the authors state that “…Si maintains its integrity.” Can this be quantified? Is this a frequency for which the film becomes stable “forever”?
8. Working mechanism…: in line 162, the authors reference a table (S4) in the supplement. Can the authors instead use the table to provide a high-level quantitative comparison of efficiency within the body text? (maybe stating something about 3 orders of magnitude lower required energy, which seems to be suggested)
9. Fast, parallel…: the various videos are really interesting providing insight into the dynamic processes of bubble formation and collapse. Can the authors provide more quantitative information regarding the refresh rate for the “animations”? Some of the videos are not real time and the frame-to-frame (or pattern-to-pattern) time seems limited by the bubble collapse time. This is not fully quantified in the paper either here or under later sections, though it can sort of be obtained from the videos. Please report some typical formation/collapse times for different sizes. Do the liquid, surface modification, and nature of the nanoparticle suspension affect these times? I am now seeing some of this in the supplement under Note 4. I think this information would be useful to include in this section of the body text (“Fast, parallel…”) as it is relevant to the refresh time for pattern generation.
10. High-precision, bubble-actuated…: this section is really interesting, and the use of different surface modifications (and potential to pattern such modifications) for different species could be very useful.
11. Microbubbles for rapid…: in line 197, the light intensity is now given in mW/cm2 (whereas earlier it was in W/cm2). Please try to be consistent so the reader can easily compare across different use cases.
12. Microbubbles for rapid…: in line 204, the bubble lifetime is discussed. Is this the sum of formation/growth and collapse times? Perhaps I missed this definition somewhere else.
13. Microbubble-platform for bioassays: in lines 234-236, a statement on metabolites is provided with examples given. It would be clearer in the parenthetical to state “(Figures 7c-d and Figures S12, S13; note that peaks associated with various metabolites shown in these figures are listed in Table S5, which provides Raman peak positions of purine derivatives identified in the literature)” or something like that. Currently, there is a bit of confusion about what the profiling data in Figures

7c-d show, so it would be good to clarify how the identified peaks correspond to various metabolites.
14. Microbubble-platform for bioassays: line 250 of the text references Table S1, which includes a significant amount of information (potentially an entire additional section of the manuscript could just be based on this table). Can the authors at least provide a high-level summary of the information in the main body text? Which advantages/disadvantages are most important? Which other methods are most prevalent, and why is the reported method superior over those most widely adopted approaches? Those answers would go a long way to establishing the high impact of the reported work.
15. Methods: in line 274, it says that "in a typical experiment, a 100 to 250 um-thick PDMS well" is used; however, in line 284, it says "using a 500 um PDMS well, as a standard condition." Is this consistent? Again, I might be missing something.
16. Methods: please use "min" as the abbreviation for minutes. Using "m" is confusing given that "m" is the abbreviation for the length unit meters.
17. Methods: was the section of CHO cell culturing/viability referenced in the text? Biocompatibility was discussed, but I cannot find reference to this work. Please clarify.

Version 1:

Reviewer comments:

Reviewer #1

(Remarks to the Author)
The authors have adequately addressed my questions.

Reviewer #2

(Remarks to the Author)
The authors have carefully addressed all my previous concerns and thoroughly revised the manuscript. In my opinion, these revisions have significantly enhanced the manuscript's quality and contribution to the field. I fully support its publication.

Reviewer #3

(Remarks to the Author)
I co-reviewed this manuscript with one of the reviewers who provided the listed reports. This is part of the Nature Communications initiative to facilitate training in peer review and to provide appropriate recognition for Early Career Researchers who co-review manuscripts.

Reviewer #4

(Remarks to the Author)
I appreciate the thorough and complete responses to reviewer comments/questions provided by the authors. I believed all previous minor concerns have been addressed, and the manuscript is now suitable for publication in Nature Communications.

Open Access This Peer Review File is licensed under a Creative Commons Attribution 4.0 International License, which permits use, sharing, adaptation, distribution and reproduction in any medium or format, as long as you give appropriate credit to the original author(s) and the source, provide a link to the Creative Commons license, and indicate if changes were made.
In cases where reviewers are anonymous, credit should be given to 'Anonymous Referee' and the source.
The images or other third party material in this Peer Review File are included in the article's Creative Commons license, unless indicated otherwise in a credit line to the material. If material is not included in the article's Creative Commons license and your intended use is not permitted by statutory regulation or exceeds the permitted use, you will need to obtain permission directly from the copyright holder.
To view a copy of this license, visit https://creativecommons.org/licenses/by/4.0/

COCKRELL SCHOOL OF ENGINEERING
THE UNIVERSITY OF TEXAS AT AUSTIN

Department of Mechanical Engineering · ETC II 5.160 · http://www.me.utexas.edu
1 University Station C2200 · Austin, Texas · 78712-0292 · (512) 471-1131 · Fax (512) 471-8727

We thank the reviewers for the positive comments. In what follows below, we have addressed all questions and suggestions and revised the manuscript accordingly. Our response can be found for each point in the following, where we also summarize the revisions we have made when updating the manuscript.

Reviewer #1 (Remarks to the Author):

General Comments: The authors describe a clever system to produce bubbles at scale in well-defined patterns using an opto-electrochemical method. The authors seem to have a good fundamental understanding of the working mechanism behind bubble formation, and they provide useful optimization results to highlight effects from reagents to lower redox voltage and surface tension toward the goal of forming bubbles with lower light intensity or with greater stability. Finally, the authors demonstrate several use cases of the bubbles to drive clustering of various colloidal particles in solution toward an example of how this system could be used in applications. Overall, the results are well-presented and described. I support publication of this work in NC, barring a few minor clarifications.

We thank the reviewer for the positive comments. We have provided the clarifications.

Specific Comments:

• The authors should elaborate on their selection of CTAC surfactant as opposed to anionic or non- ionic surfactants.

• Why does PBS perform better than hydrogen peroxide- or CTAC-only controls?

- The main purpose was to reduce surface tension and facilitate the ease with which bubbles are formed. It resulted in a lower MRLI when CTAC is added to the solution, supporting this hypothesis. We chose CTAC as it is fairly well known, and expect that both anionic and non- ionic surfactants work, but have not explored this in detail. We include this comment as well as our answer to the question regarding PBS in Supplementary Note 2, where we have added:

  "The surfactant reduces surface tension and facilitates the ease with which microbubbles are formed. We expect that other surfactants, such as anionic or non-ionic surfactants, would similarly facilitate the reduction of the MRLI. PBS was evaluated to demonstrate that the electrochemical microbubble method is compatible with solutions commonly used in cell cultures. We did not directly compare PBS with solutions containing hydrogen

peroxide or CTAC due to differences in ionic species and concentration, which would lead to different electrical impedances, making a direct comparison of electrochemical bubble behavior less meaningful. Notably, PBS has a lower overall ionic concentration than the other control solutions. To compensate for this, we applied a higher DC bias, a lower frequency, and used a thinner PDMS well. These adjustments effectively reduced the required minimum laser intensity, at times even below that needed for higher ionic strength solutions containing hydrogen peroxide or CTAC."

• Figure 2: The authors should specify in the caption or figure what the AC frequency was in 2C to which a DC bias was applied.

- We included the experimental conditions for all characterization results in the Methods section. With the helpful suggestion from the reviewer, we also added the employed electric-field condition to Figure 2's caption as follows:

"Unless stated otherwise, conditions used in characterization: 0.1 M Na_2SO_4 solution, -5 V DC / 12 Vpp at 1.5 kHz AC E-field applied for ~225 ms, and 500 μm PDMS well."

• Terminology: Do the examples of nanocrystal, EV, polymer nanosphere, and bacteria concentration really constitute assembly? Assembly in the colloidal sense usually implies some level of order amongst is being created its constituent components. I would call the structures formed aggregates (as the authors do on a few occasions), agglomerates, or clusters. Please define what is meant by assembly in this bubble-mediated context.

- We thank the reviewer for pointing out how the word is used in the field of colloidal science. We used assembly in this work for its literal meaning, "the action of gathering together as a group for a common purpose". We understand and appreciate the reviewer's concerns, and added a sentence in the introduction for clarification as the following:

  "The term "assembly" here describes primarily the action of collecting and aggregating entities without ordering them, but in some cases, we did observe the formation of more ordered semicrystalline arrangements of particles after bubble deposition."

• While the bubbles rest against the ceiling of the fluid chamber, it is not clear what happens after particle assembly. Do the complexes gain enough density to settle? If so, how does this affect the morphology of the clusters? Please discuss.

- Once the bubble shrinks to one spot, the assembled complexes attach to the surface due to electrostatic interactions and van der Waals forces. The structures adhere well enough to the surface, that they stay in place during the steps of solution removal and gentle washing. We clarified this point on page 9 of the manuscript:

"The electrochemical microbubble platform can be used to reliably deposit a variety of inorganic and biological particles, ranging from 200 nm to several micrometers in size,

onto the Si surface. The assemblies remain firmly attached, even when the surface is rinsed with DI water. The particle–particle and particle–surface adhesion can be attributed to electrostatic and van der Waals forces after bubble shrinkage (**Figure 4e**). However, when a new microbubble is formed nearby, then the assembled particle cluster can be detached and repositioned, specifically, during bubble shrinkage. Microbubbles may thus not only be used to form stable assemblies, but also be used to reconfigure the particle organization."

• Figure 7: The explanation of data in 7C-D is insufficient both in the results section and in the figure caption. In 7C (large clusters) and 7D (small clusters), there are 4 graphs with multiple measurements over time. What does each graph correspond to? Please discuss and add some level of interpretation of these results in the results section.

- Thank you for the question. Each individual graph in 7c and 7d is a different SERS measurement of bacteria assemblies created at the same condition in vancomycin solution. To avoid any possible confusion, we revised the Figure 7's caption by adding that "c) and d) show 4 representative measurement results".
- We also revised the manuscript on page 11 to clarify the above, as the following: "We successfully detect the evolving metabolite signals from assembled arrays in a prescribed manner, **as seen in the 4 representative SERS measurements in Figures 7c-d.**"

• Additionally, were control experiments performed in Figure 7 (i) without cell assembly and (ii) without vancomycin? These types of controls are crucial for understanding the benefit of this bubble- concentration approach in biological assays. This is my biggest concern with this paper.

- Yes, control experiments were performed as follows: (i) SERS measurements were performed on PS spheres without any bacterial cells, as shown in Supporting Figure 11. The peaks inherent to the Ag-on-PS spheres were excluded from the analysis of Raman peaks detected from bacterial cells. (ii) To assess metabolite release without vancomycin, we measured Raman signals from cells in the absence of the drug and found that the metabolite-related peaks remained stable for approximately 2 hours, with no noticeable decrease, as discussed in **Supporting Figure 10**.

• Did the authors apply any statistical test to Figure 7e (e.g., paired T test)?

- Yes, as discussed in the caption of Figure 7 and Supplementary Note 7, we used the Mann-Whitney U test to evaluate the statistical significance among the three sample types based on their population densities (with the dried sample having the highest density and aggregates having lower densities). This test is commonly used for small sample sizes (fewer than 30) and when the assumption of normality is difficult to verify.

Reviewer #2 (Remarks to the Author):

Summary:

The authors present an innovative opto-electrochemical method to generate highly defined microbubble patterns, enabling the assembly of nanocrystals, extracellular vesicles, polymer nanospheres, and live bacteria into complex patterns with high precision (down to 2 µm spatial resolution). Their method integrates light-based projection with low-energy electrolysis, based on a photoconducting a-Si:H surface, allowing the controlled formation of microbubbles via electrolysis upon localized illumination. By modifying the a-Si:H surface and introducing nanoparticles, they successfully suppress undesired clustering and enable individual light-addressable bubbles across a broad size range.

This work introduces a novel platform that enables dynamic, scalable, low-temperature, and high-resolution microbubble patterning - addressing major limitations of both photothermal and template-based methods. The integration of optical and electrochemical control mechanisms is a noteworthy contribution with potential for wide-ranging applications.

Originality and significance:

• The method appears innovative because it allows simultaneous generation of a large number of microbubbles and direct formation of complex shapes at room temperature, making it suitable for temperature-sensitive biological samples.

• Other methods have the limitations that the available bubble patterns are static (when prefabricated templates are used), or that the direct formation of a microbubble with a laser requires high intensities and temperatures.

This work is likely to attract considerable interest within the micromanipulation community, as the presented method addresses several longstanding limitation regarding throughput, spatial precision, and biocompatibility. The ability to dynamically project arbitrary patterns in one shot even at low power and ambient temperatures offers significant advantages in terms of scalability, versatility, and suitability for applications in biosensing and cell-based assays.

Methodology:

The experiments are well designed, executed and analyzed and strongly support the drawn conclusions. In particular, the hypothesis of an electrochemical (rather than thermal) mechanism of bubble formation is well supported.

However, the manuscript would benefit from improved clarity in certain sections, more rigorous justification of the biological proof-of-concept, and greater attention to data visualization. Specific recommendations are outlined below.

We sincerely appreciate the reviewer's thorough evaluation of our manuscript and the highly positive and accurate comments. We have addressed the questions in the improvement of the clarity as outlined below:

Detailed comments:

Clarity and context:

1. The research goal and the specific challenge being addressed were not immediately clear. A more explicit statement of the intended application early on (e.g., creation of cell-sensor assays with single- or few-cell resolution) would improve clarity. Overall strengthening of the "problem-current technological limitations-solution narrative" would enhance understanding of the potential impact and implications to a broader audience, such as that of Nat Comm.

Thank you for the helpful comments, we revised the manuscript by adding discussions related to the intended applications in the abstract and introduction as follows:

Abstract: “The microbubble-platform is highly versatile and promises new opportunities in nanorobotics, nanomanufacturing, high-throughput bioassays, bioseparation, and drug screening and discovery.”

Introduction:

“This capability is particularly useful in overcoming the technical challenges in complex device design and fabrication for high-throughput bioassays, single cell omics, and rapid drug screening. Technically, microbubble enabled particle assembly often involves the generation of a single microbubble with a focused laser beam to deposit particles onto a substrate via convective flows that form around the microbubble. The laser beam can also be raster-scanned to move the micro-bubble and thereby drive complex nanoparticle deposits.[17] However, this method is relatively slow as it involves a single microbubble and a major drawback is that it operates at elevated temperatures as the microbubbles are thermo-optically generated, which severely restricts its usefulness for any potential biological application”

2. Especially the abstract, but also the introduction could benefit from more clarity. The authors should emphasize the novelty of integrating optical and electrochemical techniques in patterning applications. Optically induced bubble patterning has been done before (studies have demonstrated the use of laser-induced microbubbles for precise material deposition ACS Applied Materials & Interfaces (this work is also cited)), and electrochemical bubble formation has also been done before, but the integration of optical and electrochemical techniques for bubble formation, which enables one shot assembly is novel.

- We thank the reviewer for the suggestion. To emphasize the novelty of our work, we have added a short summary of the work in both abstract and introduction as the following.

Abstract:

“The combination of a photoconductor with electrochemical techniques enables low-energy, low-temperature bubble generation, advantageous for large-scale, one-shot patterning of diverse particles in a biocompatible manner. The microbubble-platform is highly versatile and promises

new opportunities in nanorobotics, nanomanufacturing, high-throughput bioassays, single cell omics, bioseparation, and drug screening and discovery."

3. Justification of Biological Demonstrations and which general implications can be draw from it: The application of the platform to metabolic profiling and antibiotic response in bacteria is intriguing but insufficiently motivated. The rationale for choosing these particular biological systems is not provided. How do they showcase the unique capabilities of the platform?

Thank you for the valuable suggestion. We have revised the manuscript to better explain our choice of biological demonstration and how it highlights the platform's unique strengths:

Revision in the discussion session of the manuscript is shown as follows:

" Here, we focus on a bacteria–nanoparticle sensor array to highlight both the biological relevance and the unique strengths of our microbubble platform compared to the other technologies (**Table S3, S4**). The array enables the continuous monitoring of metabolite release from bacteria and provides biological insights into bacterial antibiotic resistance."

4. The discussion would benefit from more critical reflections on their own work? For example, how stable are the patterns? How sensitive to perturbations?

- Thank you for the suggestion. We analyzed the pattern stability and sensitivity to perturbations and added the following in the discussion section of the manuscript:

"The electrochemical microbubble platform can be used to reliably deposit a variety of inorganic and biological particles, ranging from 200 nm to several micrometers in size, onto the Si surface. The assemblies remain firmly attached, even when the surface is rinsed with DI water. The particle–particle and particle–surface adhesion can be attributed to electrostatic and van der Waals forces after bubble shrinkage (**Figure 4e**). However, when a new microbubble is formed nearby, then the assembled particle cluster can be detached and repositioned, specifically, during bubble shrinkage. Microbubbles may thus not only be used to form stable assemblies, but also be used to reconfigure the particle organization."

5. Specific lines needing clarification:

o Line 20: The term "parallel" is ambiguous. Clarify whether this refers to the simultaneous formation of multiple microbubbles as opposed to single bubble-based techniques.

- Yes, "parallel" refers to simultaneous formation of multiple bubbles. We revised the manuscript for clarity:

"This enables room temperature applications and ensures the high scalability of our technique, as a digital light projector (DLP) can project entire images and thus form

complex bubble patterns ‘in one shot’ made of many bubbles simultaneously, opposed to serial, single bubble-based techniques.”

We retained line 20 as written to ensure the abstract remains concise.

o Line 44: The transition to applications in cell-sensor assays is abrupt. Provide a smoother contextual link to explain the relevance of microbubble patterning in this domain.

- We thank the reviewer for pointing this out. We have moved the sentence to a more suitable location in the introduction so that the overall flow of the introduction is smoother. (see response below)

o Lines 49-51, 219-220: The choice of bacterial profiling as a demonstration lacks explanation. A brief rationale would improve the impact of this section.

- We thank the reviewer for the comment. In conjunction with the comment above, as well as comment 3 from reviewer #1, we have revised part of the introduction, as well as the discussion in the main manuscript so that the overall flow is smoother and why the profiling is needed.

  Introduction:
  “Finally, as a proof-of-concept, we demonstrate a microbubble-enabled nanosensor assay. The technology not only permits integration with bioassays but also enables the dynamical monitoring of bacterial cell activity in response to drugs in quasi-real time**.**”

  Main manuscript:

  “Here, we focus on a bacteria–nanoparticle sensor array to highlight both the biological relevance and the unique strengths of our microbubble platform compared to the other technologies (**Table S3, S4**). The array enables the continuous monitoring of metabolite release from bacteria and provides biological insights into bacterial antibiotic resistance.”

o Line 158-160: Clarify whether the authors recommend using only PBS for biological applications to mitigate the potential toxicity of H2O2 and CTAC.

- We thank the reviewer for the recommendation. We have added the following sentence to the manuscript:

“For biological applications, solutions such as PBS are recommended, without the addition of CTAC or H_2O_2 due to potential biocompatibility issues.”

o Line 173, Figure 4, Line 276: Specify the type, size, and concentration of nanoparticles used. Are these parameters critical to the success of the approach?

- We thank the reviewer for the comment. Upon further inspection, we updated the information. The sentence in question now reads as the following:

"The PDDA functionalization of Si further enables single-point contact of a bubble on the substrate; it also effectively helps in fixing the nanoparticles to the oppositely charged substrate (200-nm polystyrene nanospheres: 6 to 8 x 10^{10}/ml; Na_2SO_4 to 0.66 – 0.75 M) (**Figure 4d-e** and **Video S6**)."

As long as the particles remain suspended in solution, microbubbles can effectively capture and deposit them onto the substrate. We have demonstrated successful deposition of a wide range of materials with varying sizes, from 40 nm metallic silver nanoparticles, 200 nm exosomes, to micrometer-scale bacterial cells. Using high-concentration colloids improves deposition efficiency. Further optimization of particle size, type, and concentration can enhance this efficiency even more.

o Line 299: spelling error. Chinese Hamster Ovary

- Thank you for the help. We corrected the spelling.

Figures & Data Representation:

1. General comment: The current color schemes are visually overwhelming, with an excessive number of distinct colors used within single plots. A more restrained and visually coherent palette would improve both the aesthetic quality and interpretability of the figures.

- We thank the reviewer for their comment. We have edited the color scheme of Figure 7, as well as S10, S14-S16 so that the change of color at each condition is more gradual and less overwhelming.

2. Figure 4b: missing legend for the different colors and symbols of the many (10?) line plots that are displayed to show the relation between radius and time.

- Thank you for bringing this to our attention. For figure 4b, each plot represents the growth of a different bubble to show consistent behaviors. We updated the caption to clarify this point as follows:

"Results of multiple individual bubbles show consistent behaviors."

3. Figures 7c, d: Excessive color diversity without concrete meaning. Consider simplifying the palette, i.e. shades of a color to indicate time progression over 120 min or 180 min

- We thank the reviewer for the great suggestions. We revised the color theme of Figure 7 as follows :

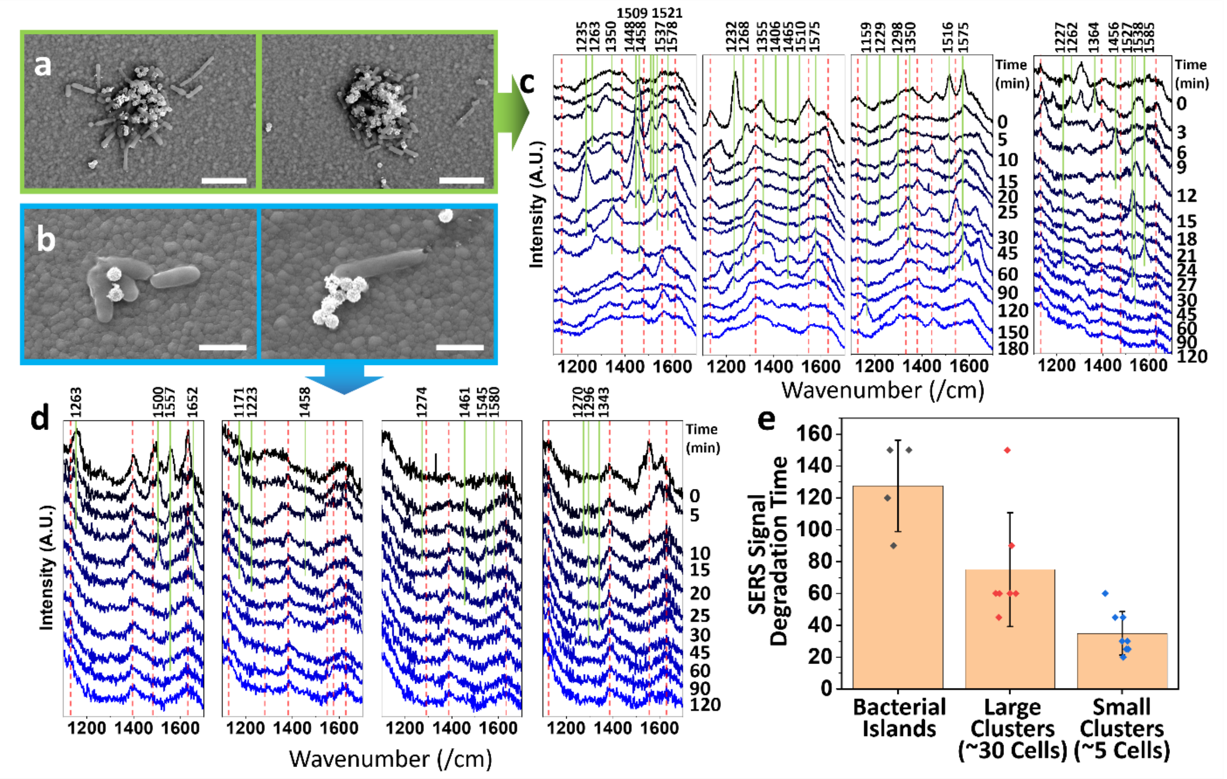

4. Figures 7c, d & S14-S16: The use of "m" for minutes is unconventional and could be confused with meters as SI unit. Replace "m" with "min" for clarity.

- We have changed “m” to “min” for clarity along with changing the color scheme of Figures S14-16 to match those displayed in Figure 7.

5. Figure S10: The insert with labels for time points (0 min, 30 min, etc.) should be larger for readability. Also, replace "m" with "min" for clarity.

- We made the suggested changes.

6. Figure S11c & S15: The meaning of different colors is unclear and should be explained.

- We added additional explanations in the captions of Figure S11 and S15:

“Each colored curve in c) is measured from a different Ag-on-PS sphere aggregate.”

And

“Colored curves in a) and b) show the radius versus time of different bubbles.”

Reviewer #3 (Remarks to the Author):

I co-reviewed this manuscript with one of the reviewers who provided the listed reports. This is part of the Nature Communications initiative to facilitate training in peer review and to provide appropriate recognition for Early Career Researchers who co-review manuscripts.

Reviewer #4 (Remarks to the Author):

The authors report an opto-electrochemical method to rapidly and controllably generate arrays of microbubbles for various purposes. Produced bubbles range in size from a few to over one hundred micrometers in size with positioning accuracy of ~2 micrometers. Biocompatibility is proven by demonstrating that the low required light intensity does not harm cells, bacteria, and sensitive nanomaterials. After fully characterizing the bubble generation process, the authors demonstrate how nanosensor-bacterial cell arrays can be used for spectroscopic profiling of metabolites to evaluate the antibiotic response of bacterial assemblies.

The authors effectively establish the need for controllable microbubble generation, primarily for acoustofluidic applications. Existing methods are presented to highlight specific shortcomings (e.g., the static nature of templates, and the thermal damage induced by conventional opto-thermal approaches). These shortcomings motivate the introduction of electrolysis for bubble production, and a creative electrode configuration (including a photoconducting alpha-Si:H layer that facilitates light- based bubble formation and growth) enables dynamic, controllable, and biocompatible microbubble arrays. The primary application of the approach appears to be for formation of particle aggregates, either nanomaterials, cells, or both and then analysis of those aggregates (as opposed to the aforementioned acoustofluidic applications). To this end, the authors provide various demonstrations, including the profiling of bacterial metabolites after exposure to antibiotics. The work is complete, the manuscript is clearly written, and the demonstrations are compelling. For these reasons, I believe the manuscript is suitable for publication in Nature Communications after addressing the mostly minor points below:

1.Introduction: as mentioned above, the alternate methods for microbubble production are primarily introduced with reference to their applications in acoustofluidics (e.g., for shaping ultrasound fields, pumping, mixing, etc.). The reported method is primarily used for nanomaterial deposition and cell aggregation. Is there an inherent difference between previous methods and the present method that makes these methods more or less suited for particular applications, or were the demonstrations chosen based on perceived greater impact? Put another way, would the current method work just as well as other methods for acoustofluidic applications?

Reply: we sincerely thank the reviewer for the thoughtful question. We do believe that light-controlled electrochemical bubbles hold strong potential for acoustofluidic applications, such as pumping, mixing, and other dynamic fluidic operations. This is an exciting direction we plan to explore in our future work. For this study, however, we chose to focus on assembling organic and biological particles. This decision was driven by the persistent challenges in biocompatibility and the difficulty of generating microbubbles in parallel, issues that have held back the use of thermally generated bubbles toward bio-applications for over a decade, despite considerable efforts across

the world in the field. Now, our method successfully addressed these limitations and demonstrated truly meaningful biological applications, opening the door to myriad biomedical research. We have highlighted this in the following statement on conventional methods to realize micro-bubbles in the introduction:

"However, this method is relatively slow as it involves a single microbubble and a major drawback is that it operates at elevated temperatures as the microbubbles are thermo-optically generated, which severely restricts its usefulness for any potential biological application."

2. Working mechanism...: the fabrication process of the enabling electrode configuration is well-described, and the method of optical activation of the local electric field is very creative. Has alpha- Si:H been used for a similar purpose (not to produce microbubbles, but just to activate the field locally)? This is purely from curiosity.

- Yes, the use of light to optically activate localized electric fields has been demonstrated in optoelectric tweezers (OET),[1] which generate patterned dielectrophoretic forces (or electroosmotic flows in subsequent work) through light-activated symmetric high-frequency AC electric fields. The working principle of DEP forces is based on leveraging interactions of electrically polarized particle and electric fields, which is entirely different from that of opto-electrochemical reactions for microbubble generation (Nature, 436, 370–372 (2005)) and we have cited the paper along with the following paragraph:

"This work, although using a similar setup to optoelectronic tweezers,[28] the mechanism is distinct, which generates bubbles and uses them for manipulation. In addition, our electrochemical bubble-based approach is fully compatible with high-ionic-strength solutions, overcoming the limitations of dielectrophoresis-based manipulation under such conditions."

3. Working mechanism...: is there an error in the text line 101? Should 7.4 W/cm2 be 0.74 W/cm2? It appears from Fig. 2b that the intensity decreases below 1000 mW/cm2 at the lowest frequency (which would be 740 mW/cm2). On that note, is there a reason for not using W/cm2 as the y-axis in Fig. 2b?

- We thank the reviewer for pointing out the error. Indeed, the value should be 0.74 W/cm^2. We have corrected this typo. The reason we used mW/cm^2 as the y axis is to assure consistency with other figures.

4. Working mechanism...: the plots in Fig. 2 have a couple of issues, i) the fonts are generally too small, particularly for the insets, which are not possible to read, ii) it might be better to have the inset for 2a as its own panel, since it is a different variable, and iii) the inset for 2b needs to make clear that the y- axis is the same as for the main plot. Generally, axes should be labeled.

- We thank the reviewer for the helpful suggestions and have made the revision of Figure 2 accordingly, as the following:

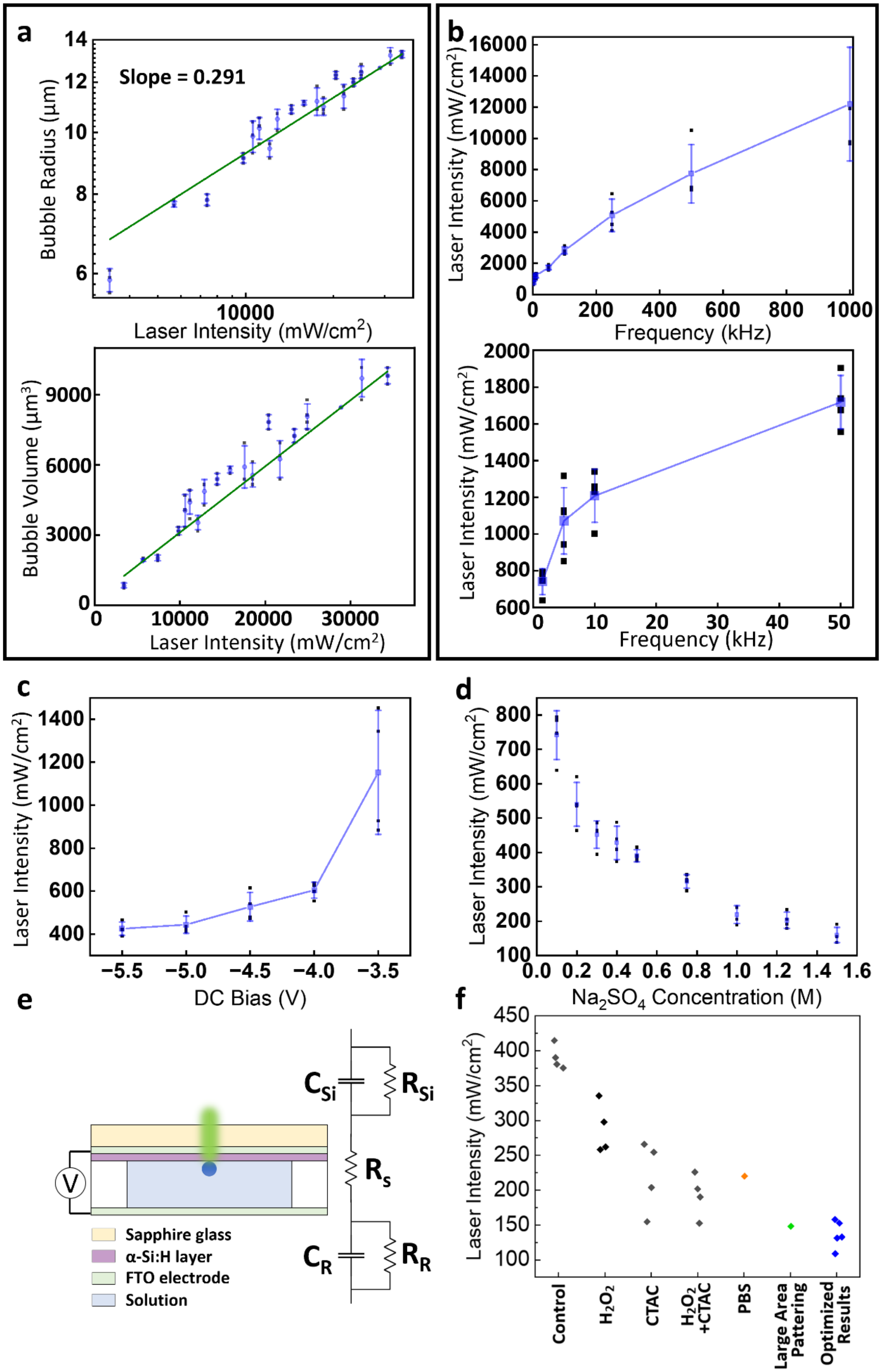

a
Slope = 0.291
Bubble Radius (μm)
Laser Intensity (mW/cm2)
Bubble Volume (μm3)
Laser Intensity (mW/cm2)
b
Laser Intensity (mW/cm2)
Frequency (kHz)
Laser Intensity (mW/cm2)
Frequency (kHz)
c
Laser Intensity (mW/cm2)
DC Bias (V)
d
Laser Intensity (mW/cm2)
Na2SO4 Concentration (M)
e
CSi
RSi
Rs
CR
RR
V
Sapphire glass
α-Si:H layer
FTO electrode
Solution
f
Laser Intensity (mW/cm2)
Control
H2O2
CTAC
H2O2 +CTAC
PBS
Large Area Pattering
Optimized Results

5. Working mechanism...: in line 103, it says "MRLI is further reduced ~2.72-fold with an increasing superimposed DC bias from -3.5 to -5.5 V". This is a bit misleading as it is not a "further" increase over the frequency dependence result. It is a separate result given that the frequency result was found at a bias of -5.0 V. Perhaps this is just word usage, but it would be clearer to say, "Further, the MRLI is reduced..." or "The MRLI is also reduced by increasing the superimposed DC bias...".

- Thank you for pointing this out. We agree that the wording is misleading and have changed it per the reviewer's suggestion to the following:

" … the MRLI is also reduced …"

6. Working mechanism...: in lines 143-144, the authors argue that the AC E-field is studied due to "its importance in understanding the working mechanism..." before mentioning robust bubble creation. The authors then spend a lot of time discussing surface degradation in DC vs. AC fields. Why is the AC E-field important to understand the working mechanism? Versus purely just avoiding degradation?

- As shown in the equivalent circuit diagram, capacitances, including those from the electric double layers, are part of the circuit diagram. When applying AC field with monotonically decreased AC frequency, we can estimate the impedance increase due to the capacitances. This effect is particularly important for electrochemical reactions occurring on the electric double layers, *i.e.*, the higher impedance, the greater voltage drop on the electric-double-layer capacitor, and the lower the limit of the laser intensity. Therefore, tuning the AC frequency and examining the associated critical limit of the laser intensity can help validate the electrochemical working mechanism. A pure DC field should be avoided, as it degrades the Si surface. Therefore, we employed an optimized AC frequency with DC bias for efficiency microbubble generation.

The above has been included in the manuscript in the following:

"Here, light turns on/off this electrochemical circuit path via controlling the Si-film's local resistance. When light is switched on and R_{Si} is significantly lowered (**Figure S3**), an applied voltage distributes across all the circuit elements, with a voltage drop proportional to each element's resistance (impedance). For a capacitor, the impedance ($Z = 1/(2\pi fC)$), and the voltage drop (V_{C_SI}), increases as the AC frequency (f) decreases. Therefore, we observe that a low AC frequency and a negative DC bias both prompt aid the HER as both effects enhance the voltage drop on the EDL capacitor

7. Working mechanism...: related to "6", in line 152, the authors state that "...Si maintains its integrity." Can this be quantified? Is this a frequency for which the film becomes stable "forever"?

We appreciate the reviewer's thoughtful question. In general, higher AC frequencies improve the stability of Si films by reducing electrochemical degradation. However, there isn't a universal frequency at which Si becomes permanently stable, since its integrity can depend on factors like film thickness, deposition method, and surface treatment. For the specific silicon films used in our study, under our applied electric field conditions, we observed stable Si performance and

achieved optimized efficiency in microbubble generation. We added the above information on page 7 of the main manuscript as the following:

“However, it should be noted that there isn’t a universal frequency at which Si becomes permanently stable, since its integrity can depend on factors like film thickness, deposition method, and surface treatment. For the specific silicon films used in our study, under the employed electric field conditions, we observed stable Si performance and achieved optimized efficiency in microbubble generation.”

8. Working mechanism...: in line 162, the authors reference a table (S4) in the supplement. Can the authors instead use the table to provide a high-level quantitative comparison of efficiency within the body text? (maybe stating something about 3 orders of magnitude lower required energy, which seems to be suggested)

- We agree that by including this information, readers may get a better general sense of energy efficiency compared to other techniques, without the need to actually check the numbers. As such, we have changed the sentence to read:

“Overall, this technique exhibits a much higher energy efficiency (>2-3 orders of magnitude) compared to other current bubble generation techniques listed in Table **S2**.”

9. Fast, parallel...: the various videos are really interesting providing insight into the dynamic processes of bubble formation and collapse. Can the authors provide more quantitative information regarding the refresh rate for the "animations"? Some of the videos are not real time and the frame-to-frame (or pattern-to-pattern) time seems limited by the bubble collapse time. This is not fully quantified in the paper either here or under later sections, though it can sort of be obtained from the videos. Please report some typical formation/collapse times for different sizes. Do the liquid, surface modification, and nature of the nanoparticle suspension affect these times? I am now seeing some of this in the supplement under Note 4. I think this information would be useful to include in this section of the body text ("Fast, parallel...") as it is relevant to the refresh time for pattern generation.

- Thank you for the comment. A more detailed analysis of the relationship between the electric field application time and the resulting microbubble size is provided in Figures 4b and 4c. Additionally, Figure 5c shows the correlation between microbubble lifetime and initial size. To avoid redundancy in explaining bubble formation and collapse, we think that the current presentation could be appropriate. The animation is intended to illustrate the tunable bubble lifetime as a demonstration, rather than for detailed analysis.

Regarding the lifetime in terms of liquid, surface modification, and the nature of the nanoparticle suspension, yes, the conditions of the solution may change the lifetime of the bubble. The shrinking of the bubble is largely mediated by 1) the dissolution of the hydrogen gas to the surrounding solution and 2) the pressure of the bubble itself coming from the surface tension. The bubble lifetime may differ depending on the initial hydrogen content, or the surface tension (which can be controlled by using surfactants such as CTAC).

10. High-precision, bubble-actuated...: this section is really interesting, and the use of different surface modifications (and potential to pattern such modifications) for different species could be very useful.

- We thank the reviewer for the comment. Indeed, by changing the surface modification, we can change the hydrophobicity of the surface, which in turn could be used for creating bubbles in a non-symmetrical shape. We envision that this may be used for creating on-demand directional flows when combined with microfluidic channels. We mentioned how the surface functionalization may change the hydrogen bubble shape and interactions with the surface in page 8.

11. Microbubbles for rapid...: in line 197, the light intensity is now given in mW/cm2 (whereas earlier it was in W/cm2). Please try to be consistent so the reader can easily compare across different use cases.

- We thank the reviewer for pointing this out. We changed the value to 0.15 W/cm^2 for consistency in revision.

12. Microbubbles for rapid...: in line 204, the bubble lifetime is discussed. Is this the sum of formation/growth and collapse times? Perhaps I missed this definition somewhere else.

- Here, lifetime is defined as the collapse time, and did not include the formation/growth time of the bubble. To increase the readability of the manuscript, we have added the definition of the lifetime as the following (in bold):

"… the bubble size and hence the bubble lifetime **(time for a bubble to collapse after growth)**…

13. Microbubble-platform for bioassays: in lines 234-236, a statement on metabolites is provided with examples given. It would be clearer in the parenthetical to state "(Figures 7c-d and Figures S12, S13; note that peaks associated with various metabolites shown in these figures are listed in Table S5, which provides Raman peak positions of purine derivatives identified in the literature)" or something like that. Currently, there is a bit of confusion about what the profiling data in Figures 7c-d show, so it would be good to clarify how the identified peaks correspond to various metabolites.

We made the revision according to your suggestion. Thank you.

14. Microbubble-platform for bioassays: line 250 of the text references Table S1, which includes a significant amount of information (potentially an entire additional section of the manuscript could just be based on this table). Can the authors at least provide a high-level summary of the information in the main body text? Which advantages/disadvantages are most important? Which other methods are most prevalent, and why is the reported method superior over those most widely adopted approaches? Those answers would go a long way to establishing the high impact of the reported work.

- We have added the following paragraph before referring to Table S1 (**Now table S3**) (instead of line 250, we add this information in the summary section – where it seems to be more appropriate as it justifies the use of our technique for metabolite detection):

“The microbubble-based assembly offers several key advantages for cell manipulation and patterning. It enables one-step trapping and attachment of cells to a substrate, without requiring the complex microfabrication typically needed for microfluidic devices, valve-based platforms, or physical traps. Additionally, the bubble-based system can capture a wide variety of particles across a broad range of sizes and properties, making it particularly effective for assembling cell/nanosensor hybrids. A detailed comparison with other state-of-the-art techniques, such as microfluidic processing,[36,37] and active cell trapping,[34,36,38] is presented in Table S3 of SI.”

15. Methods: in line 274, it says that "in a typical experiment, a 100 to 250 um-thick PDMS well" is used; however, in line 284, it says "using a 500 um PDMS well, as a standard condition." Is this consistent? Again, I might be missing something.

- The 500 um PDMS well was used only for characterization purposes and was not used in all other experiments such as the assembly of various particles, creating bubble images, and metabolite measurements. For clarity, we changed the latter sentence as the following:

“… using a 500 μm PDMS well, for characterization purposes.”

16. Methods: please use "min" as the abbreviation for minutes. Using "m" is confusing given that "m" is the abbreviation for the length unit meters.

- We have changed all instances of “m” to “min”, both in the text as well as in the figures for consistency.

17. Methods: was the section of CHO cell culturing/viability referenced in the text?

- We used CHO cells to test the viability of our bubble platform and listed the culturing methods and viability test results in the methods section. Just for the reviewer’s reference, it is mainly related to this sentence:

“The microbubble assembly exhibits excellent biocompatibility, as confirmed by standard cell-staining (Methods section).”

To enhance clarity, we revised the discussion into the following:

“The microbubble assembly exhibits excellent biocompatibility (>93%), as confirmed by standard trypan blue cell-staining of Chinese Hamster Ovary (CHO) cells after being exposed to microbubbles (Methods section).”

In addition to the changes made to the manuscript per the reviewer’s recommendations, we also noticed a typo in Figure 6, and corrected the sequence of listed scale-bar values:

“Scale bars: a) 1 μm, b) 3 μm, c) 50 μm and 25 μm in inset.”

Furthermore, we have moved the following sentence to a more suitable location for better overall flow:

“Here, we show an alternative microbubble scheme that uses low intensity light to obtain defined microbubble patterns and we show that these can be used to assemble diverse colloidal matter, including extracellular vesicles and biological cells at room temperature, into complex patterns.”

Finally, we have rearranged the Table numbers so that they match the order in the manuscript after the revision.
